# Abstracts from “Diabetic Limb Salvage: A Team Approach, 10–13th April 2024, Washington DC, USA—A joint meeting between Diabetic Limb Salvage and Wound Healing Foundation

**DOI:** 10.1111/iwj.14938

**Published:** 2024-06-18

**Laura K. S. Parnell, Gregory Schultz and John Steinberg**

The Medstar Georgetown Diabetic Limb Salvage and the Wound Healing Foundation, both nonprofit organizations, recently held a meeting on the Diabetic Foot in Washington D.C., USA. These groups chose the *International Wound Journal* to feature their submitted abstracts to highlight the excellent research ongoing in this important clinical area. If you wish to get in touch with any of the authors to discuss their research, please contact us and we can connect you.

Editorial Note: We recognize the valued contribution of Professor Greg Schultz and his connection with the IWJ to make this possible. With sadness we acknowledge his passing while attending this meeting. His legacy continues even after his passing.

## 1 ABSTRACTS

## 2 IMPACT OF METABOLIC SYNDROME ON LOWER EXTREMITY OUTCOMES IN PATIENTS WITH END STAGE RENAL DISEASE

### 2.1 **Authors:** Nichole Andrade, Georgetown University School of Medicine; Karen Li, Georgetown University School of Medicine; Christian Lava, MS, MedStar Georgetown University Hospital; Lauren Berger, Rutgers Robert Wood Johnson Medical School; Karen Evans, MD, MedStar Georgetown University Hospital

**Background:** The population of patients with End‐Stage Renal Disease (ESRD) who undergo haemodialysis continues to expand as the incidence of ESRD increases, especially in patients who are older, obese, and have multiple chronic conditions. Common comorbidities of ESRD include hypertension, diabetes, and hyperlipidemia, all of which exacerbate endothelial dysfunction and vascular disorders, placing ESRD patients at an increased risk of developing lower extremity (LE) pathology. The intersection of these comorbidities has been defined as Metabolic Syndrome (MetS). This study aims to evaluate the association between MetS in ESRD patients and LE outcomes.

**Methods:** This is a multicenter, retrospective review of all patients undergoing inpatient haemodialysis in the setting of ESRD, seen by a vascular team for the first time between January 2012 and November 2022 at four tertiary‐care hospitals within a single hospital system serving the DC and Baltimore areas. Information on patient demographics, comorbid conditions, and medical / surgical history were collected. Primary outcomes were collected up until most recent follow‐up, which included ambulatory status, amputation, and mortality.

**Results:** A total of 875 patients were included, with a total of 243 (27.8%) patients with MetS and 632 (72.2%) patients without MetS. Patients with MetS were older (63 years vs. 59 years, *p* = 1.000), had a higher BMI (28.75 kg/m2 vs 26.38 kg/m^2^, *p* = 0.994), and higher average Charlson Comorbidity Index scores (8.03⎕2.3 vs. 6.03⎕2.86, p = 1.000). There were no significant differences in history of smoking, race, or sex between the two groups. History of foot wound (37.6% vs. 13.1%, *p* < 0.000) and history of prior LE amputation (24.7% vs. 8.6%, *p* = 0.000) were both significantly higher for the MetS cohort. There were no significant differences among the history of amputation level between the two groups, although for patients with MetS, there was a higher rate of TMAs (29.6% vs. 47.5%) and a lower rate of below‐knee‐amputations (BKA) (35.4% vs. 51.9%). In our results, patients with MetS were significantly less ambulatory (86.0% vs. 93.2%, *p* = 0.001) and their levels of ambulatory status were significant (*p* < 0.001). Patients without MetS had higher rates of ambulation without assistance (89.6% vs. 77.4%). Patients with MetS also had significantly higher rates of a recent amputation (21.1% vs. 7.8%, *p* < 0.000). The levels of most recent amputation level were not significant (0.138). Rates of mortality were higher for patients with MetS (31.3% vs. 27.9%, *p* = 0.323), but not significant.

**Conclusions:** ESRD patients with Metabolic Syndrome have an almost 3‐fold increased prevalence of foot wounds, an almost 3‐fold increased prevalence of prior lower extremity amputations and an almost 3‐fold increased incidence of lower extremity amputations at follow‐up.

## 3 ORAL VERSUS INTRAVENOUS ANTIBIOTICS FOR TREATMENT OF RESIDUAL INFECTION FOLLOWING AMPUTATION IN THE DIABETIC FOOT

### 3.1 **Authors:** Jennifer Kipp, DPM, Wake Forest University School of Medicine; Lindsay LeSavage, DPM, Wake Forest Baptist Health; Joni Evans, MS, Wake Forest Baptist Medical Center; Travis Denmeade, MD, Wake Forest University School of Medicine; Cody Blazek, DPM, Wake Forest University School of Medicine

**Background:** Residual osteomyelitis is a frequent problem following surgical intervention for diabetic foot infection. The current Infectious Disease Society of America guidelines recommend 4–6 weeks of initial intravenous antibiotics for treatment of residual osteomyelitis. However, recent literature suggests oral antibiotic therapy is not inferior to intravenous therapy.

**Methods:** The primary aim of this study was to evaluate treatment success in 128 patients receiving oral versus intravenous antibiotics for residual osteomyelitis in the diabetic foot after amputation at a Level 1 academic medical trauma center. Treatment success was defined as completion of at least 4 weeks antibiotic therapy, complete surgical wound healing, and no residual infection requiring further debridement or amputation within 1 year of the initial surgery. Patients with peripheral arterial disease were excluded.

**Results:** A retrospective chart review was performed, and we found no statistically significant difference in treatment success between these two groups (*p* = 0.2766). Median time to healing for oral antibiotic treatment was 3.17 months compared to 4.06 months for intravenous treatment (*p* = 0.1045). Furthermore, there was no significant difference in group demographics or comorbidities, aside from more patients in the intravenous group having coronary artery disease (*p* = 0.0416). Type of closure and type of microbial infection was also not associated with a difference in outcomes between the two treatment arms.

**Conclusions:** The results of the present study suggest oral antibiotics for treatment of residual osteomyelitis are not inferior to intravenous therapy and may be more efficacious for certain patients regarding cost and ease of administration.

## 4 THE FIRST COMPARATIVE ANALYSIS OF PATIENT‐REPORTED OUTCOMES FOLLOWING BELOW‐KNEE VERSUS ABOVE‐KNEE AMPUTATION IN HIGH‐RISK LIMB SALVAGE PATIENTS

### 4.1 **Authors:** Danny Chamaa, MS, Georgetown University School of Medicine; Christian Lava, MS, MedStar Georgetown University Hospital; Karen Li, Georgetown University School of Medicine; Karen Evans, MD, MedStar Georgetown University Hospital; Christopher Attinger, MD, MedStar Georgetown University Hospital

**Background:** In high‐risk patients with non‐traumatic, chronic lower extremity (LE) wounds, the surgical decision for below‐knee amputation (BKA) or above‐knee amputation (AKA) is dictated by a combination of disease progression, baseline function, and patient opinion. It is well established that greater tissue preservation in LE amputations are associated with better functional outcomes compared to proximal amputations. However, patient‐reported outcome measures (PROMs) on LE function, pain, and quality of life (QoL) are limited. This study aims to compare PROMs in patients who underwent BKA or AKA for chronic LE wounds.

**Methods:** PROMs were collected via QR code for all adult chronic LE wound patients who presented to a tertiary wound center between June 2022 and June 2023. A cross‐sectional analysis of patients who underwent BKA or AKA was conducted. The 12‐Item Short Survey (SF‐12; QoL), PROM Information System Pain Intensity (PROMIS‐3a; pain), and Lower Extremity Functional Scale (LEFS; ambulatory function) were completed at 1‐month, 3‐months, 6‐months, 1‐year, 3‐years, and 5‐years postoperatively. Patient demographics, comorbidities, preoperative characteristics, and amputation details were collected.

**Results:** Of 146 survey sets, 121 (82.9%) underwent BKA and 25 (17.1%) underwent AKA.

Most patients were male (*n* = 101, 69.1%). Mean age and body mass index were 63.1 ± 0.9 years and 30.5 ± 0.7 kg/m^2^, respectively. Mean Charlson Comorbidity Index was 5.2 ± 0.2. Comorbidities for BKA and AKA patients included diabetes mellitus (71.9% vs 44.0%, respectively; *p* = 0.007), chronic kidney disease (35.5% vs. 16.0%, respectively; *p* = 0.057), and peripheral vascular disease (53.7% vs. 60.0%, respectively; 0.566). Median postoperative time point of survey completion was 9.6 (interquartile range [IQR]: 4.0–26.6) months. BKA patients had higher ambulatory function (36.1.8 ± 25.1 ± 2.3; *p* = 006), lower pain (49.7 ± 1.2 vs. 58.7 ± 2.0; *p* = 0.002), and lower psychological distress (3.3 ± 0.4 vs. 6.3 ± 0.8; p&lt;0.001) than AKA patients postoperatively. BKA and AKA patients had comparable QoL scores (29.1 ± 0.8 vs. 27.3 ± 0.9; *p* = 0.220) postoperatively.

**Conclusions:** Patients who underwent BKA for chronic LE wounds compared to AKA achieved higher functional scores, lower psychological distress scores, and comparable QoL scores. Surgical management for chronic LE wounds is multifaceted involving preoperative medical optimization, management of functional expectations, and multidisciplinary intervention to maximize PROMs.

## 5 ABSTRACT POSTERS

Poster numbers are identified in the order they were submitted.

*Identified as a top oral abstract presenter.

## 6 A COMPARATIVE ANALYSIS OF PATIENT REPORTED OUTCOMES IN NONTRAUMATIC BELOW‐KNEE AMPUTATION WITH AND WITHOUT TIBIOFIBULAR SYNOSTOSIS (ERTL)

### 6.1 **Authors:** Rachel Rohrich, MedStar Georgetown University Hospital; Karen Li, MedStar Georgetown University Hospital; Christian Lava, MS, MedStar Georgetown University Hospital; Woori Lee, Georgetown University School of Medicine; Christopher Attinger, MD, MedStar Georgetown University Hospital

**Background:** The osteomyoplastic (Ertl) approach to transtibial amputation (TTA) is an alternative to the “gold‐standard” Burgess technique that may offer a more stable residual limb and better prosthetic fit. Despite the potential for improved function, the Ertl procedure is associated with longer operative and healing times than the standard Burgess and is thus reserved for healthier and more active patients at baseline. Despite investigation in the traumatic amputation ppulation, subjective functional and quality of life (QoL) outcomes are not explored in the nontraumatic population. This study aims to compare patient‐reported outcomes measures (PROMs) in comorbid patients with chronic wounds who underwent Ertl and standard TTA.

**Methods:** A cross‐sectional review included patients who underwent either BKA with Ertl (“Ertl”) or standard Burgess BKA (“Non‐Ertl”) with postoperative follow‐ups between June 2022 and January 2024. Instruments for assessment include the 12‐Item Short Form Health Survey (SF‐12; quality of life), Patient‐Reported Outcomes Measurement Information System Pain Intensity (PROMIS‐3a; pain), Lower Extremity Functional Scale (LEFS; ambulatory function), and the Self‐Reporting Questionnaire 20‐Item (SRQ‐20; psychological distress). Data collection included demographics, comorbidities, preoperative characteristics, and operative details.

**Results:** Of 121 survey sets, 94 (77.7%) underwent Non‐Ertl and 27 (22.3%) underwent Ertl. Most patients were male (*n* = 84, 69.4%). Mean body mass index was 31.2 ± 0.7 and 29.3 ± 1.4 kg/m2 for Non‐Ertl and Ertl, respectively (*p* = 0.180). Mean age was 64.9 and 56.4 years for Non‐Ertl and Ertl, respectively (*p* = 0.004). Mean Charlson Comorbidity Index was 5.8 ± 0.3 and 3.2 ± 0.3 for Non‐Ertl and Ertl, respectively (*p* < 0.001). Comorbidities for Non‐Ertl and Ertl patients included diabetes mellitus (80.9% vs 40.7%, respectively; *p* < 0.001) and chronic kidney disease (56.4% vs. 44.4%, respectively; *p* = 0.027). Mean postoperative time points of survey completion were 15.1 ± 2.1 and 24.9 ± 3.0 months for Non‐Ertl and Ertl patients (p = 0.020). There were no significant differences observed between Non‐Ertl and Ertl patients in mean overall SF‐12 (28.6 ± 0.9 vs. 31.3 ± 1.8, respectively; *p* = 0.224) and LEFS (37.9 ± 2.0 vs. 31.0 ± 3.7, respectively; *p* = 0.105) scores. Ertl patients experienced more pain (7.2 ± 0.7 vs. 5.8 ± 0.3; *p* = 0.049) and psychological distress (5.4 ± 1.0 vs. 2.8 ± 0.4; *p* = 0.003) than Non‐Ertl patients postoperatively.

**Conclusions:** Despite the anticipated benefits of the Ertl over the standard Burgess, both approaches demonstrated similar scores for QoL and functionality. The higher pain and psychological distress reported by the relatively younger and less comorbid Ertl group may reflect a discrepancy between patient expectations and postoperative functionality. Ertl patients may foster higher functional expectations due to their healthier baseline status and the Ertl's reputation in the amputee community. However, the lack of preoperative data limits a comprehensive understanding of these findings.

## 7 A COMPARATIVE ANALYSIS OF PATIENT‐REPORTED OUTCOMES FOLLOWING FREE TISSUE TRANSFER, PARTIAL FOOT AMPUTATION, AND BELOW‐KNEE AMPUTATION IN HIGH‐RISK LIMB SALVAGE PATIENTS

### 7.1 **Authors:** Christian‐Lava, MS, MedStar Georgetown University Hospital; Karen Li, Georgetown University School of Medicine; John Steinberg, DPM, MedStar Georgetown University Hospital; Christopher Attinger, MD, MedStar Georgetown University Hospital; Karen Evans, MD, MedStar Georgetown University Hospital

**Background:** The surgical decision for limb‐salvage with free tissue transfer (FTT), partial foot amputation (PFA), or below‐knee amputation (BKA) for complex lower extremity (LE) wounds hinges on several factors, including patient choice and baseline function. However, patient‐reported outcome measures (PROMs) on LE function, pain, and QoL for chronic LE wound interventions are limited. Thus, the study aim was to compare PROMs in patients who underwent FTT, PFA, or BKA for chronic LE wounds.

**Methods:** The surgical decision for limb‐salvage with free tissue transfer (FTT), partial foot amputation (PFA), or below‐knee amputation (BKA) for complex lower extremity (LE) wounds hinges on several factors, including patient choice and baseline function. However, patient‐reported outcome measures (PROMs) on LE function, pain, and QoL for chronic LE wound interventions are limited. Thus, the study aim was to compare PROMs in patients who underwent FTT, PFA, or BKA for chronic LE wounds.

**Results:** Of 200 survey sets, 71 (35.5%) underwent FTT, 51 (25.5%) underwent PFA, and 78 (39.0%) underwent BKA. Median postoperative time points of survey completion between FTT (6.8 months, IQR: 23.1), PFA (11.1 months, IQR: 15.1), and BKA (6.2 months, IQR: 21.3) patients were comparable (*p* = 0.8672). Most patients were male (*n* = 92, 76.0%) with an average age and body mass index (BMI) of 61.8 ± 12.6 years and 30.3 ± 7.0 kg/m^2^, respectively. Comorbidities for FTT, PFA, and BKA patients included diabetes mellitus (DM; 60.6% vs. 81.6% vs. 67.3%; *p* = 0.165), peripheral vascular disease (PVD; 48.5% vs. 47.4% vs. 42.3%; *p* = 0.790), and chronic kidney disease (CKD; 12.1% vs. 28.8% vs. 42.1%; p = 0.084). No significant differences were observed between BKA, PFA, and FTT patients in mean overall PROMIS‐3a T‐scores (49.6 ± 14.8 vs. 54.2 ± 11.8 vs. 49.6 ± 13.7; *p* = 0.0976), LEFS scores (37.5 ± 18.0 vs. 34.6 ± 18.3 vs. 38.5 ± 19.4; p = 0.4567), or SF‐12 scores (28.0 ± 8.6 vs. 29.5 ± 2.9 vs. 29.0 ± 4.0; *p* = 0.298).

**Conclusions:** Patients receiving FTT, PFA, or BKA for chronic LE wounds achieve comparable levels of LE function, pain, and QoL postoperatively. Patient‐centered functionally‐based surgical management for chronic LE wounds using interdisciplinary care, preoperative medical optimization, and proper patient selection optimizes postoperative PROMs.

## 8 A NATIONWIDE VETERANS HEALTH ADMINISTRATION OBSERVATIONAL COHORT STUDY COMPARING THE RATE OF LOWER EXTREMITY AMPUTATIONS IN VETERANS HOSPITALIZED WITH AND WITHOUT COVID‐19

### 8.1 **Authors:** Aliza Lee, DPM, MS, SVAMC; Tanvi Patil, PharmD, SVAMC; Venita Cucurella Smith, DPM, SVAMC; Devene Prince, PharmD, SVAMC

**Background:** The COVID‐19 pandemic lockdown impacted the ability of veterans to receive timely access to care. Previous studies signal towards increased risk of major amputations for patients with critical limb ischemia and diabetic foot ulcers. This study aims to compare the rate of amputation in veterans hospitalized with COVID‐19 with those without COVID‐19.

**Methods:** This nationwide, retrospective, observational cohort study included veterans aged 18 years or older hospitalized with COVID‐19 from January 1st, 2020, through January 1st, 2022 using a nationwide Veterans Health Administration (VHA) database. The COVID‐19 group consisted of patients hospitalized with COVID‐19 who had a positive test within 7 days of hospitalization. The control group consisted of patients hospitalized for reasons other than COVID‐19 without a prior positive test or during the study duration. The primary outcome was comparing the rates of all lower extremity amputations between patients hospitalized with COVID‐19 versus those hospitalized without COVID‐19 infections. The secondary outcome was to compare the rates of all amputations in a predefined subgroup of patients with diabetes. Propensity scores (PS) constructed using logistic regression informed by pre‐identified variables meeting the disjunctive cause criterion was used for 1:1 matching. Cox proportional hazards regression models were used to estimate hazard ratios (HRs) and 95% confidence intervals (CI) and adjusted for pre‐specified confounding variables. Outcomes were identified using validated ICD‐10‐CM‐based algorithms.

**Results:** This study included 48 786 patients in both the matched cohorts. The average patient population was 69 years old, predominantly white (~72%) males(~94%). Baseline covariates were well‐balanced after PS matching. Incidence rates for lower extremity amputation were 1.80 versus 1.75 per 100 person‐years among patients hospitalized with COVID‐19 versus those hospitalized without COVID‐19 infections. The adjusted (a)HR for lower extremity amputation was 1.26 (95% CI 1.12–1.41). Incidence rates for lower extremity amputation in patients hospitalized with COVID‐19 versus without were 0.47 versus 0.49 per 100 person‐years in patients without diabetes and 3.46 versus 3.35 per 100 person‐years in diabetic patients. The aHR for lower extremity amputation was 1.27 (95% CI 1.12–1.43) and 1.05 (95% CI 0.78–1.42) in subgroup of patients with and without diabetes.

**Conclusions:** Overall, this study shows that patients hospitalized with COVID‐19 19 may increase the rate of lower extremity amputations compared to those hospitalized without COVID‐19 however this seems to be primarily driven by subgroup of patients with diabetes.

## 9 A RETROSPECTIVE COHORT REVIEW OF DEEP AND SUPERFICIAL DÉBRIDEMENT TECHNIQUES FOR LOWER EXTREMITY SPLIT THICKNESS SKIN GRAFTING

### 9.1 **Authors:** Rachel Rohrich, MedStar Georgetown University Hospital; Karen Li, Georgetown University School of Medicine; Christian Lava, MS, MedStar Georgetown University Hospital; Sami Alahmadi, MS, Georgetown University School of Medicine; Christopher Attinger, MD, MedStar Georgetown University Hospital

**Background:** Patients with non‐healing lower extremity (LE) wounds often require a split thickness skin graft (STSG) for closure. Nonviable tissue must be debrided thoroughly prior to STSG inset. Our study aims to compare differences in debridement depth on STSG outcomes for non‐healing LE wounds.

**Methods:** 442 LE wounds in 307 patients who underwent STSG from December 2014 to December 2022 were reviewed. Wounds that received superficial debridement (SD) were compared to wounds that received deep debridement (DD), defined by debriding to the level of white tissue underlying the granulation tissue. Skin graft loss was compared between groups.

**Results:** Overall, 266 (60.2%) wounds received SD and 176 (39.8%) wounds received DD. Patients in the SD group demonstrated a higher median Charlson Comorbidity Index (5 vs. 4, *p* = 0.070) and significantly higher prevalence of diabetes mellitus (DM), neuropathy, chronic kidney disease (CKD), history of myocardial infarction (Hx MI), and congestive heart failure (CHF). Median wound surface area was 30.0 (IQR: 68.0) cm2, and similar between groups (*p* = 0.365). Positive debridement cultures prior to STSG were comparable between the DD (77.6%) and SD (83.2%) groups (*p* = 0.222). The DD cohort showed a trend towards lower STSG loss of any kind (19.3% vs. 27.1%, *p* = 0.062), while demonstrating significantly lower rates of full graft loss (7.4% vs. 16.9%, *p* = 0.004) and reduced postoperative infection (7.4% vs. 13.5%, *p* = 0.044). After adjusting for significant comorbidities (DM, CKD, CHF, Hx MI, BMI, smoking) as well as pre‐debridement sterility and acellular dermal matrix use in a multivariate logistic regression, DD was independently associated with decreased odds of full graft loss (OR: 0.2, CI: 0.3, 0.9, *p* = 0.003) and any graft loss (OR: 0.5, CI: 0.1, 0.6, *p* = 0.027). Subgroup analysis revealed that DD significantly decreased full graft loss in non‐sterile wounds (5.8% vs. 17.3%, *p* = 0.007), but did not in sterile wounds (8.8% vs. 16.1%, *p* = 0.227).

**Conclusions:** The depth of debridement to remove unhealthy tissue is important to achieve successful outcomes and high rates of graft take. Our results show that the deep debridement technique is a viable method and should be considered to effectively manage chronic, non‐sterile LE wounds.

## 10 *APPLICATION OF EXTRACORPOREAL CIRCULATION COMPRESSION PERFUSION IN THE TREATMENT OF DIABETIC FOOT: A RETROSPECTIVE CROSS‐SECTIONAL STUDY

### 10.1 **Authors:** Leio Gao, MD, PhD, Beijing Shijitan Hospital Affiliated Capital Medical University; Jiangning Wang, MD, PhD, Beijing Shijitan Hospital Affiliated Capital Medical University

**Background:** Diabetic foot is a serious complication of diabetes. Infection and peripheral artery disease (PAD) often lead to lower limb amputation. During surgical microcirculatory vascuar reconstruction of the diabetic foot, we used Extracorporeal circulation compression perfusion(ECCP) to achieve circulatory perfusion of ischemic limbs under high perfusion pressure, and promote the reconstruction of the peripheral vascular network during perfusion.

**Methods:** We retrospectively evaluated 113 patients with diabetic foot admitted from January 2018 to August 2021.The patients were grouped according to whether they received ECCP treatment; experimental group: 31 patients, controls: 82 patients. After applying the inclusion criteria and exclusion criteria, there were 29 patients in the experimental group and 57 patients in the control group. Foot microcirculation was evaluated by measuring the percutaneous oxygen partial pressure (TcPO2),infrared thermography (IRT) and CT angiography(CTA). Wound healing time and ulcer recurrence rate 6 months after discharge were compared between the groups.

**Results:** TcPO2 and IRT values in the experimental group differed significantly compared with the control group. Foot ulcer healing time in the experimental group was shorter than that in the control group (16.22 ± 3.08 days vs 27.51 ± 4.52 days, respectively), and the recurrence rate after 6 months in the experimental group was lower than that in the control group (6/29, 20.6% vs. 27/57, 47.3%, respectively).

**Conclusions:** ECCP can accelerate wound healing speed and reduce ulcer recurrence. ECCP expand the stenosis site of the diseased vessel, increase the blood supply to the tissue by increasing blood flow through the cross section of the vessel per unit time.

## 11 ARTHRODESIS TO MAXIMIZE THE FUNCTIONALITY OF THE VERTICAL CONTOUR CALCANECTOMY: A NOVEL CONSIDERATION

### 11.1 **Author(s):** Tiffanie Liu, DPM, MedStar Georgetown University Hospital; Ali Fadel, DPM, MedStar Georgetown University Hospital; Craig Verdin, DPM, UT San Antonio; Christopher Ply, MS, Georgetown University School of Medicine; Jayson Atves, DPM, MedStar Georgetown University Hospital

**Background:** Amputation of any level of the lower extremity has biomechanical and functional consequences but in the presence of limb threatening entities, amputations may be necessary for the purpose of overall limb preservation (1, 2). Heel ulcerations with concomitant calcaneal osteomyelitis are difficult to heal due to the lack of soft tissue in this area as well as limited choices for surgical resolution. The heel is the least common location for foot ulceration, yet it carries the highest rate of morbidity and mortality. As many as 52% of patients undergo major amputation due to calcaneal osteomyelitis (3–5). A partial calcanectomy, alternative to below‐knee‐amputation (BKA) has been shown to be an acceptable intervention for limb preservation (7–14).

5‐year mortality rate greatly increases for patients who become non‐ambulatory. Patients unable to walk after amputation have a 30.1% survival rate while patients who maintain ambulatory status have a 68.8% survival rate (15). If the patient is ambulatory at baseline, the goal of limb salvage should be to return to baseline status. Losing a portion of the weight‐bearing surface of the calcaneus places patients at increased risk for abnormal limb function and ambulation, residual deformity, wounds, infection, and amputation (6,13,16). The Achilles tendon plays a significant role in gait by providing proprioception and propulsion. In the setting of calcaneal osteomyelitis, the Achilles is often infected and its resection results in rearfoot instability. Loss of ambulation and function can be as high as 24% (16).

**Methods:** At our institution, a vertical contour calcanectomy (VCC) is performed as opposed to the traditional partial calcanectomy (11). Once infection is managed and the surgical incision has healed, there may be residual deformity and instability. With the goal to return to baseline function, tendon balancing and/or bony fusion can be performed as a secondary procedure to address the resulting weakness and instability. The power of the tibiotalocalcaneal (TTC) arthrodesis has been recognized in other conditions and deformities as a means to retain lower extremity function and patient independence (17–19). In the appropriate patient, a TTC can be a valuable tool for the purpose of limb salvage.

We propose that the TTC fusion can be used to address the long‐term sequelae of deformity, instability, and gait dysfunction after VCC. By addressing ankle instability after loss of the Achilles and a portion of the weight bearing surface of the calcaneus, the patient's quality of life can be improved by a TTC fusion to increase function and return the patient to baseline ambulation, avoiding the mortality and major amputation rate that is associated with the conventional partial calcanectomy alone (4, 16).

This study provides our surgical outcome and post‐operative gait data to quantitatively substantiate the effectiveness of TTC arthrodesis after partial calcanectomy for the maximization of functional limb salvage and restoration of gait in one patient. Using gait analysis and lower extremity functional scale scores, we examined functional outcome measures.

**Results:** 55yo male with latent autoimmune diabetes in adults and peripheral neuropathy presented with a heel wound (Figure 1). It had been present for 9 months, developing after elective tendo‐Achilles lengthening. No clinical signs of acute infection noted. Biomechanical exam revealed 20 degrees of ankle dorsiflexion past neutral (Figure 2). The ulcer was due to the patient's calcaneal gait, likely a consequence of Achilles over‐lengthening. It was discussed with the patient to undergo an Achilles shortening procedure to correct the calcaneal gait and allow the wound to heal.

Before the planned surgery, the patient presented to the emergency department. The ulcer progressed to a malodorous wound with purulent drainage and visible bone, concerning for calcaneal osteomyelitis. XRs revealed radiolucency with destructive changes and periosteal reaction on the inferior calcaneus (Figure 3). CT confirmed fluid collection and osseous changes to the calcaneus, consistent with osteomyelitis and soft tissue infection.

Source control of the infection was obtained by an incision and drainage and partial calcanectomy. The Achilles tendon's distal aspect was excised from its attachment and there was significant resection of the posterior calcaneal tuber (Figure 4). Intraoperative cultures resulted with clean margins and the patient returned for a definitive VCC with primary closure (Figure 5).

The patient progressed without incident and returned to activity 53 days after closure (Figure 6). The patient expressed difficulty ambulating due to ankle instability, requiring the use of a walker. Restoration of function was imperative and he elected to proceed with TTC arthrodesis.

65 days after VCC, TTC fusion was performed to provide rearfoot stability and a more functional limb. An anterior ankle plate was utilized with screws spanning both ankle and subtalar joints.

Postoperatively, protected ambulation began at 3 months, with progression to full weight bearing in diabetic shoes and lace‐up ankle brace at 4 months. He returned to daily activities with no restrictions at 6 months and has continued to maintain unassisted weightbearing at 15 month followup.

6 months after TTC arthrodesis, the patient consented to gait analysis. Using wearable sensors, the patient completed a 120‐second walk test and a 30‐second Romberg test that evaluated 11 gait parameters to compare to normal gait values. Temporal–spatial data are summarized in Table 1.

**Conclusions:** Heel wounds have the highest rate of morbidity and mortality relative to other foot wounds. Heel wounds with concomitant calcaneus osteomyelitis only increases the risk of continued infection, chronic wounds, and risk of amputation (5). A partial calcanectomy, although an acceptable limb salvage procedure, is associated with increased functional morbidity and deformity, with the residual limb at risk for major amputation, which has a rate of loss of ambulation that can be as high as 69.1% (25). Even without major amputation, there is concern for decreased limb function after a partial calcanectomy. The tibiotalocalcaneal (TTC) arthrodesis has been recognized as a powerful tool to address dysfunctional biomechanics, especially in patients with deformity and partial foot amputations, and should be considered as a reconstructive technique in ambulatory patients with gait instability after partial calcanectomy.

As with any procedure, patient selection is critical and involves a thorough history and physical examination in addition to understanding the patient's functional expectations and demands. We understand that not every patient is a candidate for reconstruction, however in the properly selected patient, we contend that the partial calcanectomy, or VCC, with a TTC arthrodesis can potentially increase the functionality of the residual limb.

This case report highlights the potential benefit of a tibiotalocalcaneal arthrodesis by increasing lower extremity functionality after a healed ipsilateral partial calcanectomy. The TTC arthrodesis addresses the rearfoot instability caused by the Achilles tendon resection that often occurs during a partial calcanectomy or vertical contour calcanectomy. This lower limb reconstruction allows the patient to better return to baseline functionality as evidenced by the resulting postoperative LEFS score and gait study data. Discussion about the patient's daily activities and ambulatory status prior to reconstruction and the functional expectations after the TTC arthrodesis is necessary for selecting surgical candidates. This case report allows for further comparative analysis studies regarding patient postoperative functional morbidity and mortality between VCC alone and with a subsequent TTC arthrodesis to increase rearfoot stability in ambulatory patients.

## 12 ASEPTICALLY PROCESSED HUMAN RETICULAR ACELLULAR DERMAL MATRIX CAN SUPPORT LIMB PRESERVATION IN PATIENTS WITH LARGE TISSUE DEFECTS WITH LATE STAGE DERMATOPOROSIS

### 12.1 **Author:** Charles Marchese, DPM, Manalapan Foot & Ankle

**Background:** Deep dissecting hematoma (DDH) is a major complication of dermatoporosis that can be limb threating. Multimodality treatment aimed at providing what the wound lacks and to reconstruct tissue is often necessary. Often a large zone of necrosis and tissue loss cannot be treated by primary closure or secondary intention and requires skin substitutes to preserve the limb. The application of dermal matrix substitute can provide a scaffold with matrix proteins and replace the damaged integument. Aseptically processed human reticular acellular dermal matrix (HR‐ADM) provides an open, uniform scaffold with preserved matrix proteins that support cell infiltration and integration, that are crucial for epithelialization and tissue formation. The aetiology, diagnosis and treatment are discussed in a case series illustrating the vital role of HR‐ADM in limb preservation.

**Methods:** The deep hematomas were debrided carefully, resulting in large tissue defects. The HR‐ADM was applied and secured in place. Standard treatment protocols were followed until complete closure was achieved.

**Results:** Despite challenging patients, graft integration was observed and wound closure. The HR‐ADM provided the missing dermal structure that was required following the deep dissection of the hematoma. The dermal structure is vascularized and incorporated, culminating in full closure and limb salvage.

**Conclusions:** With the aging population, DDH is an emerging clinical entity with an increased prevalence. It can be a major potentially lethal complication of dermatoporosis. HR‐ADM provides the missing dermal framework that is remodelled and integrated to replace the damaged tissue and preserve the limb.

## 13 *CAREGIVER FATIGUE OF LOWER EXTREMITY AMPUTEES

### 13.1 **Authors:** Eleanor Dunlap, DNP, University of Maryland Medical Center; Suzanna Fitzpatrick, DNP, University of Maryland Medical Center; Khanjan Nagarsheth, MD, University of Maryland Medical Center

**Background:** With the increase in health care costs and decrease in home care services nationwide, health care is shifting from hospital based care to community based care which is often provided by family. Caregiver Fatigue (also known as caregiver strain or burden) is defined as the strain or load borne by a person who cares for a chronically ill, disabled, or elderly family member. This is a multidimensional concept that includes both optimistic and pessimistic aspects of providing care, and can lead to psychological pain, physical health issues, financial and social strains, impaired family relationships, a sense of hopelessness, and other negative outcomes of caring tasks. As the population in the US continues to grow, so does the number of people living with chronic disease, with more than half (51.5%) of adults having a diagnosis of a chronic disease. Specifically, one subset of patients with a cluster of chronic diseases such as PAD, DM, ESRD who progress to needing lower extremity amputations, which in turn, leads to a different type of ongoing care and support. In a large urban academic vascular outpatient clinic, it was noted that caregivers of the amputation population may need additional support to help both them and the patient have optimal results following amputation.

**Methods:** A convenience sample of 10 lower extremity amputation patients who presented with a caretaker over a 3 month period were given two surveys related to caregiver fatigue. The Zarit Burden Interview tool and the Short Form Zarit Burden Interview (ZBI‐12), which are validated surveys used to assess the effects of care on caregivers. In addition to collecting the survey data for the patient and caretaker, demographic data was obtained.

**Results:** Out of the 10 patients, 7 were male and 3 female. Age range was 42–73 years (mean 61.5). Patients had a past medical history of PAD 70% (7/10), HTN 90% (9/10), DM 80% (8/10), CAD 40% (4/10), and ESRD 30% (3/10). The contralateral limb was previously amputated in 2 patients, had vascular intervention due to atherosclerotic disease in 3 patients, and 4 patients had known atherosclerotic disease which was being monitored. The caregivers were wives in 6 patients, daughters in 2 patients, the husband in 1 patient, and son in 1 patient. On the Zarit Burden Interview survey, the patients felt that their caretakers were fatigued with a mean score 32.1 (range 22–53) which indicated mild to moderate burden while the caretakers reported a mean score of 43 (range 19–58) indicating moderate to severe burden. On the Short Form Zarit Burden Interview (ZBI‐12) the patients felt that their caretakers were fatigued with a mean score 15 (range 0–26) which indicates mild to moderate burden while the caretakers reported a mean score of 21.6 (range 12–31) indicating high level of burden.

**Conclusions:** Caregivers can have a moderate to high level of burden when caring for a family member with lower extremity amputation. While the amputee recognizes that there is some strain from needing care, they do not assess the level of burden. Family, community and social support are important aspects to decrease the strain/buden/fatigue of caregiving and may not be offered to all patients. Organizations that offer emotional support and counselling, and community day care centers can help ease the caregiver's burden by allowing the caregiver to take adequate rest and recuperate.

## 14 COMPARATIVE OUTCOMES OF TWO CELLULAR TISSUE‐BASED PRODUCTS IN GUILLOTINE TRANSMETATARSAL AMPUTATION: A RETROSPECTIVE STUDY IN CLTI PATIENTS

### 14.1 **Authors:** Tyler L. Coye, MD, Baylor College of Medicine; Alejandro Zulbaran Rojas, MD, Baylor College of Medicine; Miguel Bargas Ochoa, MD, Baylor College of Medicine; Bijan Najafi, PhD, Baylor College of Medicine; Jayer Chung, MD, Baylor College of Medicine

**Background:** This study aims to compare the effectiveness of two cellular/tissue‐based products (CTPs), Integra and Hyalomatrix, for wound healing in patients with chronic limb‐threatening ischemia (CLTI) undergoing guillotine Transmetatarsal Amputations (gTMA).

**Methods:** The study involved a retrospective analysis of demographic data and wound healing outcomes in CLTI patients undergoing gTMA followed by either Integra or Hyalomatrix CTP placement. Key variables included the proportion of healed gTMAs, the rate of split thickness skin graft (STSG) placement, and the average wound healing times from CTP placement. A survival analysis was conducted to determine time to heal between groups.

**Results:** 48 patients were included (*n* = 20 Integra vs. *m* = 28 Hyalomatrix). There were no significant differences in clinical characteristics between groups. There were no significant differences in the proportion of healed gTMAs (Integra 65% vs. Hyalomatrix 50%; *p* = 0.31) or in wound healing time after CTP placement (Integra 245.3 vs. Hyalomatrix 207.9 days; *p* = 0.46). However, the proportion of patients requiring STSG placement was significantly higher for those undergoing Integra placement (80% [*n* = 16/20]) compared to Hyalomatrix (39% [*n* = 11/28; *p* = 0.01]). Kaplan Meier analysis revealed no statistically significant difference in healing times between the two treatment groups.

**Conclusions:** The study concludes that wound healing outcomes between Integra and Hyalomatrix are similar in CLTI patients with gTMAs, however, with a higher need for STSG for aiding wound healing in the Integra group. This research provides valuable insights for clinical decision‐making in treating CLTI patients undergoing gTMA.

## 15 DIABETIC NEUROPATHY AND FUNCTIONAL GAIT CHALLENGES: EXPLORING GAIT DIFFERENCES IN PATIENTS WITH AND WITHOUT PERIPHERAL NEUROPATHY WITHIN A DIABETIC POPULATION

### 15.1 **Authors:** Meghan Currin, Georgetown University School of Medicine; Christopher Attinger, MD, MedStar Georgetown University Hospital; Jayson Atves, DPM, MedStar Georgetown University Hospital; John Steinberg, DPM, MedStar Georgetown University Hospital; Karen Evans, DPM, MedStar Georgetown University Hospital

**Background:** Peripheral neuropathies (PN) are a broad range of disorders affecting the peripheral nervous system commonly associated with diabetes mellitus (DM). As the disease progresses, patients may experience decreased mobility, altered gait, and ulceration that ultimately lead to loss of function. As a consequence, these patients may undergo non‐traumatic lower extremity (LE) amputation. Early identification of gait abnormalities in PN patients may aid in optimizing LS outcomes, specifically pertaining to postoperative healing and prevention of re‐ulceration in the future. This study aims to analyse spatiotemporal gait parameters of DM patients with and without PN.

**Methods:** Patients were prospectively enrolled between June 2021 and January 2024 if they were (1) =18 years old, (2) able to safely and independently ambulate without pain or assisted devices, (3) had no open lower extremity (LE) wounds, and (4) no LE surgeries within the last 3 months. Patient demographics, comorbidities, preoperative characteristics, and amputation details were collected. Participants completed a protocol including wearable sensors, a 120‐second walk test, and a 30‐second Romberg (sway) test. The Motility Lab software (Hamilton Thorne) provided data on cadence, gait speed, elevation midswing, single limb support, double limb support, stride length, and sway. Data analysis used an independent sample *t*‐test (*p* < 0.05, STATA VSN 18.0). A multivariable logistic regression analysis including chronic kidney disease (CKD) and peripheral vascular disease (PVD) was performed to control for confounding variables.

**Results:** Of the 452 patients completing spatiotemporal gait testing, 185 (69.2%) patients had DM. Of whom, 128 (30.8%) had PN (PN+) and 57 (30.8%) did not have PN (PN‐). Mean age was 63.50 ± 10.89 years. Mean body mass index (BMI) was 31.80 ± 8.06 kg/m^2^. PN+ patients had significantly higher rates of CKD (*n* = 32, 29.1% vs. *n* = 8, 15.1%; *p* < 0.001) and PVD (*n* = 50, 39.1% vs. *n* = 8, 14.0%, *p* = 0.001). There were more males in the PN+ cohort than the PN‐ cohort (*n* = 89, 69.5% vs. *n* = 28, 49.1%; *p* = 0.008). PN predicted slower gait speed (0.81 ± 0.23 vs. 0.89 ± 0.21; *p* = 0.018) and cadence (97.12 ± 11.64 vs. 100.81 ± 11.33; p = 0.027), lower single limb support (35.47 ± 3.13 vs. 36.98 ± 0.33; p = <0.001), and increased double limb support (29.30 ± 5.71 vs. 26.12 ± 4.77; *p* ≤ 0.001), shorter stride length (0.99 ± 0.22 vs. 1.05 ± 0.20; *p* = 0.031), and increased sway (0.21 ± 0.18 vs. 0.16 ± 0.10; *p* = 0.016). Multivariate regression revealed PN decreased single limb support (ß = −1.02, *p* = 0.033) and increased double limb support (*ß* = 2.53, *p* = 0.005).

**Conclusions:** PN significantly impacts gait parameters of patients with DM, indicating a deterioration in their walking patterns. Recognizing the early signs of gait abnormalities can aid in timely interventions to minimize the risk of ulcers and further complications, underscoring the importance of a multidisciplinary approach to patient care.

## 16 DOES LENGTH OF STAY IMPACT PATIENT‐REPORTED OUTCOMES MEASURES IN NON‐TRAUMATIC LIMB SALVAGE PATIENTS UNDERGOING FREE FLAPS?

### 16.1 **Authors:** Christian Lava, MS, MedStar Georgetown University Hospital; Karen Li, Georgetown University School of Medicine; Rachel Rohrich, MedStar Georgetown University Hospital; Christopher Attinger, MD, MedStar Georgetown University Hospital; Karen Evans, MD, MedStar Georgetown University Hospital

**Background:** The management of non‐traumatic lower extremity (LE) limb salvage patients undergoing free tissue transfer (FTT) is complex and necessitates a multidisciplinary approach. The duration of hospitalization may reflect the surgical complexity, complications, and patient care burden, potentially influencing their recovery. However, its impact on patient‐reported outcome measures (PROMs)—key to evaluating functionality, satisfaction, and quality‐of‐life (QoL)—remains understudied. This study aims to investigate the influence of length of stay (LOS) on PROMs in limb salvage patients undergoing FTT procedures.

**Methods:** A single‐center cross‐sectional study was conducted, including patients who underwent FTT, with a postoperative visit between June 2022 and January 2024. Length of stay (LOS) was categorized as hospital (i.e., difference between admission and discharge dates) and postoperative (i.e., difference between date of surgery and discharge dates). The 12‐Item Short Survey (SF‐12; QoL), PROM Information System Pain Intensity (PROMIS‐3a; pain), and Lower Extremity Functional Scale (LEFS; ambulatory function) were completed at multiple times points: 1‐month, 3‐months, 6‐months, 1‐year, 3‐years, and 5‐years postoperatively. Patient demographics, comorbidities, preoperative characteristics, and amputation details were collected.

**Results:** A total of 41 patients underwent FTT, totalling 86 survey sets. Median postoperative duration of survey completion was 6.4 (interquartile range [IQR]: 2.4–25.5) months. The majority were male (*n* = 33, 74%). Mean age and body mass index were 60.2 ± 14.0 years and 29.9.9 ± 5.7 kg/m^2^, respectively. Mean Charlson Comorbidity Index (CCI) was 4.1 ± 2.7. Comorbidities included diabetes mellitus (*n* = 23, 56.1%), peripheral vascular disease (*n* = 21, 41%), and chronic kidney disease (*n* = 6, 14.6%). Mean hospital and postoperative LOS were 28.5 ± 12.2 and 14.6 ± 7.2 days, respectively. Patients with a longer postoperative LOS showed reduced ambulatory function (*r* = −0.247; *p* = 0.048), although this was not observed with hospital LOS (*r* = 0.1566, *p* = 0.609). Both hospital and postoperative LOS were associated with increased pain postoperatively (*r* = −0.390, *p* = 0.002 and *r* = −0.332, *p* = 0.007, respectively). QoL did not show significant differences based on either hospital LOS (*r* = 0.1566, *p* = 0.609) or postoperative LOS (*r* = −0.075; *p* = 0.808).

**Conclusions:** This study demonstrates that LOS following FTT procedures significantly influences PROMs, including ambulatory function and pain intensity, which may be multifactorial. Collecting PROMs over time with larger sample sizes is critical for enhancing our understanding of patient experiences in the face of limb loss.

## 17 *DR. ChatGPT? CURRENT APPLICATIONS AND EFFICACY OF ARTIFICAL INTELLIGENCE IN DIABETIC FOOT ULCER CARE

### 17.1 **Authors:** Rachel Rohrich, MedStar Georgetown University Hospital; Christian Lava, MS, Medstar Georgetown University Hospital; Karen Li, Georgetown University School of Medicine; Isabel Snee, Georgetown University School of Medicine; Christopher Attinger, MD, MedStar Georgetown University Hospital

**Background:** Diabetic foot ulcer (DFU) care and surgical management represent a significant challenge in plastic and reconstructive surgery. The rise of artificial intelligence (AI), notably ChatGPT, represents a resource for DFU patients to seek information regarding their care. In attempts to qualify its utility as a patient resource, we evaluated the accuracy, comprehensiveness, and safety of ChatGPT responses to frequently asked questions (FAQs) related to DFU care.

**Methods:** 11 DFU care FAQs were posed to ChatGPT Model 3.5 in December 2023. Questions were divided into topic categories of Wound Care, Concerning Symptoms, and Surgical Management. Four plastic surgeons in our wound care department evaluated responses for quality on a 10‐point Likert scale for accuracy, comprehensiveness, and danger, defined by the extent to which closely following ChatGPT‐3.5's advice could harm patients. Department attending provided qualitative feedback. Response readability was assessed using 10 different readability indexes.

**Results:** Overall, ChatGPT answered patient questions with an mean accuracy of 8.7 ± 0.3, comprehensiveness of 8 ± 0.7, and danger of 2.2 ± 0.6. Averaged across all readability metrics, ChatGPT answered at mean grade level of 11.9 ± 1.8. Qualitatively, physician reviewers complimented the “comprehensiveness and simplicity” of the responses (*n* = 11/11) and the AI's ability to provide “generally good” information (*n* = 4/11). While only three responses were noted to present explicitly incorrect information, the majority of responses (*n* = 10/11) left out key information, such as DVT symptoms and comorbid conditions impacting salvage.

**Conclusions:** We observed that ChatGPT may provide misinformation, omit crucial details, and respond at nearly 4 grade levels higher than the American average, warranting caution for DFU patients and providers alike. However, ChatGPT was sufficient in its ability to provide general information, which may allow DFU patients to make more informed decisions, be more comfortable with their care, and learn how to prepare for their recovery. The utility of ChatGPT is likely to be further integrated into clinical practice and consultations. It is becoming increasingly synonymous with the future of healthcare and to keep pace with emerging trends, physicians should be prepared to talk about AI with their patients. Physicians must bridge the gap between potential benefits of ChatGPT and the current limitations, as seen in our study.

## 18 EVALUATING FUNCTIONALITY OF MULTIPLE LEVELS OF NON‐TRAUMATIC AMPUTATION THROUGH KINEMATIC GAIT PARAMETERS

### 18.1 **Authors:** Emery Steinberg, MedStar Georgetown University Hospital; Holly Shan, Georgetown University School of Medicine

**Background:** The level of amputation for patients with non‐traumatic foot pathologies is a complex decision that limb salvage surgeons face, with ambulation playing a crucial role in the quality of life and long‐term survival of amputees. In recent years, gait sensors have been used to assess ambulation. This study investigates functional outcomes of digit, ray, transmetarasal (TMA), and below‐knee amputations (BKA) using wearable gait sensor.

**Methods:** Between December 2021 and January 2024, adult patients who were able to ambulate unassisted, without experiencing pain, and who did not have an open wound or had undergone lower extremity surgery in the preceding 3 months, were eligible for participation. Patients who had undergone amputations at various levels, including digit amputation, ray resection, TMA, or BKA, were included in this analysis. Patient characteristics, preoperative factors, and surgical history were collected. Participants completed a standardized assessment protocol using wearable gait sensors. This involved completing a 120‐second walk test at a self‐selected pace. The data collected from the sensors were analysed using the Motility Lab software, with statistical significance set at *p* < 0.05.

**Results:** A total of 77 patients were included for analysis, comprising 30 (38.9%) digit, 12 ray (15.6%), 12 TMA (15.6%), and 23 BKA (29.9%). The average age (61.31.19 years), body mass index (30.80.71 kg/m^2^), and Charlson Comorbidity Index (4.90.2) did not differ between groups. Proportion of smokers (*n* = 30, 34.8%), diabetes mellitus type II (*n* = 54, 62.8%), chronic kidney disease (*n* = 24, 29.1%), end stage renal disease (*n* = 7, 8.1%) was similar across all four groups. There were no differences in gait speed (0.800.02 m/s, *p* = 0.414), elevation mid‐swing (1.620.14 cm, *p* = 0.063), step duration (0.630.01 s, *p* = 0.066), cadence (96.21.2 steps/min, *p* = 0.434), single limb support (34.40.3%, *p* = 0.185), double limb support (29.20.6%, *p* = 0.089), and stride length (0.970.02 m, *p* = 0.225) across level of amputations.

**Conclusions:** Our study shows comparable ambulatory function across all levels of amputation. This may highlight the importance of selecting the right amputation for each patient, the multidiscipinary limb salvage approach and improvements in wearable aids. We encourage other large limb salvage centers to utilize wearable sensors to assess functionality of non‐traumatic amputations.

## 19 EVOLUTION OF DIABETIC WOUND FROM BENIGN TO MALIGNANT

### 19.1 **Authors:** Liliya Parkman, DPM, MedStar Georgetown University Hospital; Tiffany Hoh, DPM, MedStar Georgetown University Hospital; David Martin, MD, MedStar Georgetown University Hospital

**Background:** Diabetes mellitus presents a significant challenge with its array of complications, notably chronic wounds, which impose a substantial burden on both patients and healthcare systems. Although diabetic wounds are commonly perceived as benign, it is increasingly recognized that they can undergo transformation into malignant lesions. This case highlights the transformation of a seemingly “typical” diabetic wound into a malignancy, emphasizing the critical importance of vigilance and the reinforcement of the necessity for biopsy.

Case Study: 61 year old male with a history of type II diabetes, smoking, and osteomyelitis of bilateral hands. He originally presented in April of 2022 with a necrotic right heel wound, a low grade fever, leukocytosis with strep bacteremia, thus septic. His pathology came back positive for calcaneal osteomyelitis and necrotic tissue. For the next 9 months he was treated surgically and conservatively with IV antibiotics and wound care. He was hospitalized multiple times and his pathology consistently returned the same. Overall his wound has clinically improved during this time span. In July of 2023 his wound exam showed abnormal filamentous skin appearance at its base, concerning for a wart versus malignancy. A biopsy was taken and came back as atypical squamous proliferation described as Plantar verrucous carcinoma also known as ‘epithelioma cuniculatum’ He was recommended for further evaluation with plastic surgery and oncology. A repeat biopsy was completed showing the same result.

**Methods:** Procedures: Surgically, this patient underwent a partial calcanectomy, multiple debridements, skin grafts, wound excision, closure with a z‐plasty, revascularization. Conservatively, he underwent treatment with IV antibiotics and wound care modalities including Aquacel silver, wet to dry dressings, and negative pressure wound therapy.

**Results:** A regular diabetic wound transformed to malignant lesion detected by in office biopsy when clinical changes noted on clinical exam.

**Conclusions:** Several factors contribute to diabetic wound malignancy, including chronic inflammation, tissue damage, oxidative stress, and dysregulated cell proliferation which foster an environment conducive to oncogenic mutations. Early detection and intervention are critical to prevent diabetic wound malignancy. Regularly monitoring chronic wounds for abnormal growth or inadequate response to treatments is vital. Histopathological examination and molecular profiling help detect pre‐malignant changes, guiding appropriate management. The potential for diabetic wounds to become malignant underscores the need for vigilance and comprehensive management. Further research is necessary to understand the molecular mechanisms and develop effective preventive and treatment strategies for diabetic wound malignancies.

## 20 *FACTORS INFLUENCING PATIENT‐INITIATED COMMUNICATION IN BELOW‐KNEE AMPUTATION FOR DIABETIC CHRONIC LOWER EXTREMITY WOUNDS: A PRELIMINARY ANALYSIS OF 88 CASES

### 20.1 **Authors:** Isabel Snee, Georgetown University School of Medicine; Rachel Rohrich, MedStar Georgetown University Hospital; Christian Lava, MS, MedStar Georgetown University Hospital; Karen Li, Georgetown University School of Medicine; Christopher Attinger, MD, MedStar Georgetown University Hospital

**Background:** Patients undergoing below‐knee amputation (BKA) for diabetic complications often experience heightened anxiety and poor psychosocial outcomes perioperatively. Patient‐initiated‐communication (PIC) has been utilized to address unclear information, but may also lead to burnout among surgeons and office staff. Our study aims to characterize patient factors associated with increased PIC in this patient population.

**Methods:** We retrospectively reviewed patients receiving BKA from December 2021 to August 2023. PIC in the form of phone calls and portal messages documented in the perioperative period (defined as ±90 days of surgery) were reviewed. PIC patients were compared to patients that did not initiate PIC (non‐PIC). We further conducted a subgroup analysis of patients who initiated preoperative PIC (pre‐PIC) and postoperative PIC (post‐PIC). Primary outcomes were (1) incidence of PIC within the perioperative period, (2) rationale for PIC, and (3) characteristics associated with PIC patients.

**Results:** A total of 88 patients underwent BKA, 65 (73.9%) of whom initiated communication during the perioperative period, for a total of 206 communication encounters in the perioperative period. The cohort was primarily male (*n* = 59, 67.1%) and the mean age was 57.9 ± 13.3 years with a mean Charlson Comorbidity Index (CCI) of 5.0 ± 2.8. Pre‐PIC (*n* = 111, 53.9%) was more common than post‐PIC (*n* = 95, 46.1%). Collectively, the majority of PICs were administrative (*n* = 73, 35.4%). Pre‐PIC was related to medication, (*n* = 20, 18.0%), wound care (*n* = 18, 16.2%), wound symptoms (*n* = 13, 11.7%) and procedural details (*n* = 7, 6.3%), and post‐PIC concerned medication (*n* = 25, 26.3%) and wound care (*n* = 14, 14.7%). There were no differences in age (*p* = 0.464), gender (*p* = 0.345), or CCI (*p* = 0.382) between patients that engaged in PIC and those that did not. Overall, PIC patients demonstrated a higher rate of psychiatric history other than major depressive and generalized anxiety disorders (38.7% vs. 0.0%, *p* = 0.011) compared to non‐PIC patients. Subgroup analysis revealed that pre‐PIC patients were more likely to be married (61.3% vs. 38.6%, *p* = 0.033) than non‐pre‐PIC patients. Post‐PIC patients were more commonly discharged to home rather than a rehabilitation facility (28.6% vs. 10.3%, *p* = 0.047) and experienced less complications requiring a return to the OR 30 days postoperatively (6.1% vs. 24.3%, 0.026).

**Conclusions:** Perioperative PIC is prevalent among BKA patients at our institution. Identifying reasons for PIC and high‐incident patient groups can guide quality improvement efforts to proactively address patient concerns and reinforce resources already established by our institution, such as amputee support groups and prosthetist consultations.

## 21 FILLET FLAP COVERAGE FOR CLOSURE OF DIABETIC FOOT AMPUTATIONS: A RETROSPECTIVE REVIEW OF 70 PATIENTS

### 21.1 **Authors:** Elonay Yehualashet, Georgetown University School of Medicine; Christian Lava, MS, MedStar Georgetown University Hospital; Karen Li, Georgetown University School of Medicine; Karen Evans, MD, MedStar Georgetown University Hospital; Christopher Attinger, MD, MedStar Georgetown University Hospital

**Background:** In the United States, 2.4–4.5 million individuals suffer from chronic lower extremity (LE) wounds, with a rising incidence due to an aging population, diabetes mellitus (DM), peripheral vascular disease (PVD), and obesity. The fillet flap (FF) leverages the “spare parts” algorithm in reconstructive surgery–utilizing tissue from amputated or otherwise non‐salvageable body parts, thus avoiding donor‐site morbidity. However, the efficacy of FF for minor LE amputations due to chronic LE wounds remains understudied. This study aims describes our institutional experience with FF in high‐risk patients with chronic LE wounds.

**Methods:** A retrospective review of patients undergoing foot amputation with FF coverage for chronic LE wounds between January 2013 to August 2023 was conducted. Patient characteristics, operative details, and postoperative outcomes were collected. Primary outcome was FF survival, defined as no necrosis =7 days postoperatively. Secondary outcome was complications, categorized into short‐ (<30 days postoperatively) and long‐term (=30 days postoperatively).

**Results:** A total of 70 patients were included for analysis. Mean age and body mass index (BMI) was 65.0 + 13.7 years and 28.8 ± 6.4 kg/m2, respectively. In 45 patients (64.2%) with preoperative angiography, the patency rates were: first dorsal metatarsal artery (*n* = 10, 22.2%), lateral plantar artery (*n* = 7, 15.6%), medial plantar artery (*n* = 6, 13.3%), and dorsalis pedis artery (*n* = 4, 8.9%). Mean follow‐up duration was 9 (IQR: 32) months. Fourteen (20.0%) patients experienced short‐term complications, including re‐ulceration (*n* = 7, 10.0%), deep surgical site infection (SSI; i.e., abscess, gangrenous necrosis; *n* = 6, 8.6%), and superficial SSI (i.e., cellulitis, *n* = 4, 5.7%). Eleven (15.7%) patients necessitated reoperation for debridement (*n* = 4, 5.7%), wound closure (*n* = 4, 5.7%), flap necrosis (*n* = 3, 4.29%), incision and drainage (ID; *n* = 1, 1.4%), and/or split‐thickness skin grafting (STSG; *n* = 1, 1.4%), foreign body exploration (*n* = 1, 1.4%). Twenty‐eight (40.0%) patients experienced long‐term complications, including re‐ulceration (*n* = 21, 28.6%), deep SSI (*n* = 16; 22.9%), superficial SSI (*n* = 5, 7.1%), and hematoma formation (*n* = 1, 1.4%). Thirty‐two (52.89%) patients necessitated reoperation for debridement (*n* = 16, 22.9%), ID (*n* = 8, 11.4%), STSG (*n* = 6, 8.6%), wound closure (*n* = 5, 7.1%), hematoma evacuation (*n* = 1, 1.4%), and/or removal of heterotopic bone (*n* = 1, 1.4%). Collectively, FF survival was 90% (*n* = 63).

**Conclusions:** FF facilitates reconstruction in complex cases and should be integrated into each chronic LE wound algorithm to avoid additional donor‐site morbidity, and to facilitate stump‐length preservation or limb salvage.

## 22 GAIT PARAMETERS FOLLOWING NON‐TRAUMATIC MINOR AND MAJOR AMPUTATIONS: A COMPARATIVE ANALYSIS FOR FUNCTIONAL LIMB SALVAGE

### 22.1 **Author:** Jie Jung Shih, MS, Georgetown University School of Medicine

**Background:** Limb salvage surgeons are often faced with choosing between a transmetatarsal amputation (TMA) and below‐knee amputation (BKA) when there is unsalvageable foot pathology. Although TMA, a lower level amputation that has greater potential for return to ambulation, the chances of reoperation to a higher level of amputation is greater, thus exposing a patient already with high comorbidities to more high‐risk surgeries. This study investigates functional outcomes of ray amputations, TMA, and BKA through comparative gait analysis.

**Methods:** From December 2021 to July 2023, adult patients who could safely ambulate unassisted and without pain, without open wound, and without previous lower extremity surgery in the previous 3 months were offered participation. Patients with ray amputation, TMA, or BKA were included in this analysis. Charleston Comorbidity Index (CCI) scores were found through chart review and level of amputation were verified through x‐ray review. Participants completed a standardized protocol with wearable sensors, completed a 120‐second walk test at a self‐selected pace, and a 30‐second Romberg (sway) test. We analysed data provided from the Motility Lab software. Data was analysed using ANOVA with significance defined as *p* < 0.05.

**Results:** Of the 368 patients in the gait database, 67 patients received an amputation (Ray, *n* = 38; TMA, *n* = 14; BKA, *n* = 15). 2/15 (13.3%) of BKA patients had a prior TMA. BKA patients were younger than Ray and TMA (55.69 8.55 vs. 66.12 10.29 vs. 64.63 12.34 years) but had similar BMI and CCI Scores. There were no differences in gait parameters across all amputation levels in gait speed, elevation mid‐swing, step duration, cadence, single limb support, double limb support, and sway.

**Conclusions:** Ambulation is important for quality of life in limb salvage patients, and no clear guidelines exist in on how to preserve functionality based on level of amputation. To the authors' knowledge, this is the first study to use gait as a measure of functionality to compare multiple levels of amputation. Gait parameters did not differ between patients receiving TMA and BKAs.

## 23 HINDFOOT ALIGNMENT EFFECT ON MIDFOOT CHARCOT RECONSTRUCTION OUTCOMES

### 23.1 **Authors:** Liliya Parkman, DPM, MedStar Georgetown University Hospital; Thomas Milisits, DPM, MedStar Georgetown University Hospital; Christopher Attinger, MD, MedStar Georgetown University Hospital; John Steinberg, DPM, MedStar Georgetown University Hospital; Nicole Cates, DPM, MedStar Georgetown University Hospital

**Background:** Charcot Neuroarthropathy is a progressive disease affecting the bones and joints of neuropathic patients, characterized by tissue destruction. The leading theory attributes Charcot Neuroarthropathy to an inflammatory response triggered by repetitive trauma, disrupting bone remodelling factors. This leads to joint destruction and bone resorption, resulting in foot and ankle deformity, potentially leading to ulceration and osteomyelitis. While abnormal hindfoot biomechanics contribute to midfoot stress, there's limited literature on the specific impact of hindfoot alignment on midfoot Charcot Neuroarthropathy outcomes. Research suggests that a varus hindfoot moment arm increases the risk of naviculocuneiform and tarsometatarsal joint breakdown, while a valgus hindfoot moment arm increases the risk of midtarsal joint breakdown. The study aims to evaluate the effect of hindfoot alignment on 210 patients with midfoot Charcot Neuroarthropathy who underwent Charcot reconstruction. Preoperative hindfoot alignment was categorized into valgus, neutral, and varus using the Saltzman view.

**Methods:** Inclusion criteria encompassed adults over 18 with midfoot Charcot, who underwent reconstruction from 2004 to 2021. Preoperative hindfoot alignment was categorized into valgus, neutral, and varus using the Saltzman view. Hindfoot alignment was based on the most distal part of the calcaneus location in relation to the longitudinal axis of the tibia, neutral is in line, varus is medial, and valgus is lateral. Patients' hindfoot alignment was assessed for its effects on midfoot charcot reconstruction. Multivariate logistic regression evaluated the effect of hindfoot alignment on reconstructive outcomes.

**Results:** In patients with valgus position, the odds of pin tract infection were 4.2 times higher (4.237, 95% 1.165–15.406), new site of Charcot collapse was 4.9 times higher (4.920, 95% 1.093–22.156), hardware failure was 3.5 times higher (3.454, 95% X 1.260–9.468) than neutral. The odds of osteomyelitis was 5.3 times higher (5.319, 95% 1.156–24.390) in valgus than varus. In patients with varus position, the odds of pin tract infection were 5.8 times higher (5.750, 95% 1.538–21.496), osteomyelitis was 2.8 times higher (2.758, 95% 1.017–7.477) than neutral.

**Conclusions:** Varus and valgus hindfoot alignment propagated worse complications than neutral in midfoot Charcot. Hindfoot malalignment in patients who underwent reconstruction may stimulate further Charcot breakdown and post‐operative infection including hardware failure, pin tract infections, and osteomyelitis. Evaluation of hindfoot alignment is important to mitigate postoperative complications in midfoot Charcot reconstruction.

## 24 *HYPOTHERMIC STORAGE PRESERVES NATIVE TISSUE CHARACTERISTICS AND FUNCTIONS AS A PROTECTIVE BARRIER AND SCAFFOLD

### 24.1 **Authors:** Katrina Harmon, PhD, Organogenesis; MaryRose Kammer, MS, Organogenesis; Justin Avery, PhD, Organogenesis; Kelly Kimmerling, PhD, Organogenesis; Katie Mowry, PhD, Organogenesis

**Background:** Placental membranes contain extracellular matrix (ECM) proteins, growth factors, and cytokines, and have been shown clinically to support wound closure 1,2. A proprietary processing technique has been developed using a hypothermic storage processing technique to retain native characteristics of amnion (HSAM°) and chorion (HSCM†) membranes. In this study, hypothermically stored amnion and chorion were evaluated for their ability to maintain key native characteristics in vitro.

**Methods:** Maintenance of matrix structure as well as ECM and regulatory proteins were evaluated using scanning electron microscopy (SEM) and immunohistochemistry, respectively. In vitro scaffold functionality was assessed using a simulated wound fluid (SFW) degradation model, and changes were evaluated through mass loss and SEM. AlamarBlue and imaging were utilized to evaluate human dermal fibroblast attachment and proliferation for 7 days.

**Results:** HSAM and HSCM maintained key structures of unprocessed placental membranes. Collagens I and II, HGF, IGF‐1, and TGF‐ß1 were maintained in hypothermically stored membranes. HSAM retained intact epithelial, stromal, and spongy layers, while HSCM retained the reticular, basement membrane, and trophoblastic layers. HSAM and HSCM withstood rapid degradation for over 17 days, with tissue loss and retained structures comparable to unprocessed membranes. Additionally, HSAM and HSCM supported attachment and proliferation of dermal fibroblasts.

**Conclusions:** These studies highlight maintenance of key characteristics using hypothermic storage of amnion and chorion membranes.

## 25 IMMEDIATE AMBULATION AFTER MAJOR LOWER EXTREMITY AMPUTATION USING AN EXTERNAL FIXATOR DESIGN IMMEDIATE POSTOPERATIVE PROSTHESIS

### 25.1 **Authors:** Christopher Bibbo, DO, Sinai Hospital of Baltimore; Asma Jappar, DPM, Veterans Affairs Medical Center

**Background:** Over 185 000 below knee amputations performed/year in United States. Improved post‐operative mobility, simultaneous wound care ability, and enhanced time final prosthesis is desirable. The authors describe the use of a novel immediate external fixation based immediate postoperative prosthesis (X‐Prosthesis®, PostOp Innovations, Fallston, MD) in 18 multi‐morbid patients, including diabetics, allowing full weight‐bearing, simultaneous soft tissue care and improved time to final prosthesis.

**Methods:** Timing of X‐Prosthesis® and amputation; Full Weight Bearing: PACU (*n* = 3, 18%) [time point zero]; POD #1 = 61% (*n* = 11). POD #2 (*n* = 2, 11%), POD #3 (*n* = 1) or POD #4 (*n* = 1). Mean time full weight‐bearing with X‐Prosthesis® = 1.3 days. Mechanical device complications: loose half‐pin (*n* = 2); fine wire discomfort (*n* = 2); wire failure (*n* = 1) BMI = 69 + disuse tibial osteopenia) [one super obese patient (BMI = 76) five‐ring construct had no external fixation complications]. Medical Complications &amp; Peri‐Operative Deaths: none.

**Results:** Final Prosthetic/Functional Outcomes: Mean time to final prosthesis = 3.3 weeks (3.32 months) [literature reference = 18.4 weeks (4.6 months)]. Final median functional ambulation grade = K3 (two grade K level improvement). Patient Satisfaction: overall 94% satisfaction, would undergo surgery with X‐Prosthesis® again for contralateral limb/recommend to another patient.

**Conclusions:** This study demonstrates that the X‐Prosthesis® in multi‐morbid patients is valuable to enhance amputee rehabilitation by immediate weight‐bearing and allow continued care of soft tissues.

## 26 IMMEDIATE VERSUS DELAYED SKIN GRAFTING OF FREE MUSCLE FLAPS FOR LIMB SALVAGE: DOES TIMING MATTER?

### 26.1 **Authors:** Sabrina DeLeonibus, MS, Georgetown University School of Medicine; Karen Li, Georgetown University School of Medicine; Christian Lava, MS, MedStar Georgetown University Hospital; Richard Youn, MD, MedStar Georgetown University Hospital; Karen Evans, MD, MedStar Georgetown University Hospital

**Background:** Free muscle flaps (FMF) require split‐thickness skin grafts (STSG) for coverage. Medically comorbid patients undergoing FMFs have demonstrated surprisingly high rates of skin graft failure over the FMF. This study therefore characterizes risks for STSG failure and the effect of staging STSG on graft outcomes in medically comorbid patients.

**Methods:** A retrospective review of patients undergoing STSG for LE FMF coverage between 2011 and 2023 was performed. Demographics, comorbidities, FMF details, STSG details, and complications were collected. Primary outcome was graft failure.

**Results:** Ninety‐one patients underwent FMF and STSG with 65 (71.4%) undergoing immediate STSG and 26 (28.6%) undergoing delayed, at a median of 12 days (IQR = 9) after FMF. The delayed group had a significantly higher Charlson Comorbidity Index (5.6 vs. 3.7, p&lt;0.001). Overall graft failure rate was 31.5%, with no differences between groups (immediate: 27% vs. delayed 42.3%, *p* = 0.157). On multivariate analysis, elevated preoperative HbA1c (OR:1.5, CI = 1.1–1.9), low levels of albumin preoperatively (OR:0.3, CI: 0.1–0.9), and history of Charcot arthropathy (OR:8.6, CI:1.3–55.2) were independent predictors of graft failure.

**Conclusions:** Little evidence exists to help guide the decision to perform immediate versus delayed skin grafting of FMFs in highly comorbid population undergoing limb salvage. Delaying skin grafts in patients with significant comorbidities that threaten flap viability and wound healing capacity may improve graft take. Patient comorbidities, nutritional status, and intraoperative factors should also be considered when determining the timing of skin grafts over FMF.

## 27 IMPACT OF POSTOPERATIVE LENGTH OF STAY ON PATIENT‐REPORTED OUTCOMES AFTER NON‐TRAUMATIC BELOW‐KNEE AMPUTATION

### 27.1 **Authors:** Christian Lava, MS, MedStar Georgetown University Hospital; Karen Li, Georgetown University School of Medicine; Rachel Rohrich, MedStar Georgetown University Hospital; Karen Evans, MD, MedStar Georgetown University Hospital; Christopher Attinger, MD, MedStar Georgetown University Hospital

**Background:** In chronic lower extremity (LE) wound patients facing below‐knee amputations (BKA), several factors influence recovery and long‐term outcomes. The duration of hospitalization may reflect the surgical complexity, complications, and patient care burden, potentially influencing their recovery. Patient‐reported outcome measures (PROMs) are tools utilized to measure patients' own experience of their health such as mobility, mental health, and social function. This study aims to investigate the influence of length of stay (LOS) on PROMs in limb salvage patients undergoing BKA procedures.

**Methods:** A single‐center cross‐sectional study was conducted, including patients who underwent BKA, with a postoperative visit between June 2022 and January 2024. Length of stay (LOS) was categorized as hospital (i.e., difference between admission and discharge dates) and postoperative (i.e., difference between date of surgery and discharge dates). The Lower Extremity Functional Scale (LEFS; ambulatory function) and 12‐Item Short Survey (SF‐12; quality of life [QoL]) were completed at multiple times points: 1‐month, 3‐months, 6‐months, 1‐year, 3‐years, and 5‐years postoperatively. Patient demographics, comorbidities, preoperative characteristics, and amputation details were collected.

**Results:** A total of 50 patients underwent a BKA, totalling 94 survey sets. Median postoperative duration of survey completion was 6.9 (interquartile range [IQR]: 3.2–20.4) months. The majority were male (*n* = 37, 74%). Mean age was 60.8 ± 12.9 years. Mean body mass index (BMI) was 30.3 ± 6.5 kg/m2. Median Charlson Comorbidity Index (CCI) was 4 (IQR: 3–6). Comorbidities included diabetes mellitus (*n* = 37, 74.0%), peripheral vascular disease (*n* = 22, 44%), and chronic kidney disease (*n* = 16, 32.0%). Median hospital and postoperative LOS were 8 (IQR: 5–12) and 7 (IQR: 4–11) days, respectively. By =6 months, patients with a longer postoperative LOS experienced lower ambulatory function (*r* = −0.2522; *p* = 0.017). QoL did not differ significantly according to postoperative LOS (*r* = 0.133; p = 0.600).

**Conclusions:** Postoperative LOS following BKA procedures significantly influences patient‐reported ambulatory function. Further PROM studies are crucial for assessing BKA interventions, informing patient‐centered decision‐making.

## 28 *IMPACT OF TREATMENT WITH HIGH CONCENTRATION CAPSAICIN (8%) TOPICAL SYSTEM ON SENSORY TESTING IN PATIENTS LIVING WITH PAINFUL DIABETIC PERIPHERAL NEUROPATHY OF THE FEET: A POST‐HOC ANALYSIS OF THE PACE TRIAL

### 28.1 **Authors:** Kent Gordon, PhD, Averitas Pharma; Michael King, DPM, Upperline Health; Samuel Allen, PhD, Averitas Pharma; Audrey Carnevale, PhD, Averitas Pharma; Nathanial Katz, MD, WCG Analgesic Solutions

**Background:** The most prevalent complication of diabetes is neuropathy, occurring in 50% of patients, which typically manifests as a loss of distal sensory function in the lower extremities.^1^ The likelihood of diabetic peripheral neuropathy (DPN) increases with the duration of diabetes and at least one out of five patients experience pain.^1‐3^

The vascular supply to peripheral nerves in patients with DPN is often inadequate, leading to compromised blood flow resulting in distal axons that are innately too weak to support themselves for the long transport of nutrients, neurotrophic factors, as well as other signals.^4^ DPN represents a state of bioenergetic failure and impaired metabolism, resulting in significant nerve damage. Additionally, although DPN is progressive, the disease course is punctuated by attempted small nerve fibre regeneration, which ultimately succumbs to net degeneration and underscores the crucial need for developing effective, broad‐spectrum therapies.^5^ These consequences of DPN can result in physical and chemical alterations of epidermal nerve fibres and change sensation in the extremities.^1,6^ As patients combat both the loss of mechanical sensory information and increasing perception of pain, quality of life (QOL) can be directly impacted1.

PACE was an open label, Phase III, randomized, 3‐arm, multinational safety, and tolerability study in patients with painful DPN over 52 weeks. The study was designed to assess the safety and effect of 30‐minute repeat applications of QUTENZA (capsaicin) 8% topical system (QTZ) with standard of care (SOC) vs. SOC alone.^7,8^ The primary endpoint was the change from baseline in the Norfolk quality of life diabetic neuropathy (QOL‐DN) total score, which is a validated patient‐reported outcome questionnaire.^7^ This assessment was specifically developed to reliably measure effects of nerve function that translate into changes in QOL, activities of daily living, and health of the individual.

**Methods:** Although the a priori hypothesis of PACE was to assess potential deterioration of sensory function in the QTZ + SOC group, the observed reduction in the QOL‐DN total score suggested a measurable improvement in patients with DPN (Figure 1 [insert Figure 5 from Vinik 2016 minus QTZ 60 min group])7. This current post‐hoc analysis was conducted to further analyse changes in sensory function within the QTZ + SOC group after multiple applications using a more detailed secondary measure. The Brief Sensory Pain Examination was used to identify clinically relevant deficits in sensory function at baseline and to re‐assess the same following repeated applications of QTZ. These results must be interpreted with caution as this is a post‐hoc analysis of an open‐label study.

The Brief Sensory Pain Examination assessed reduced or increased stimulus perception by rating evoked sensations from the most painful area compared to an asymptomatic control site. It involved the assessment of 5 modalities: vibration, heat, cold, sharp sensations, and the assessment of deep tendon reflexes. Treatment area was identified based on the presence of spontaneous and evoked pain (includes mechanical dynamic allodynia [e.g., pain in response to brush stroke], cold or warm thermal allodynia [e.g., pain in response to touch with a cold or warm object], and/or hyperalgesia [e.g., exaggerated pain in response to pinprick] as determined by a sensory examination from the investigator. Although multiple locations on the foot were examined, for the purpose of this publication all data included are from the mid‐plantar location. These data seemed most consistently representative of the entire foot. Examinations were performed by neurologists as well as non‐neurologist physician‐investigators given study training and findings were captured using pre‐defined scales (Table 1).

The post‐hoc analysis included two subgroups of patients. The first subgroup was comprised of patients with a reported score of zero (no sensation) at baseline in each of the five modalities. The second subgroup was comprised of patients with abnormally low sensation reported at baseline in each of the five modalities. The percentage of sensory tests in these two groups that shifted to either some positive, or normal sensation, respectively, were collected in patients treated with QTZ for 30 min + SOC.

**Results:** Due to the open label nature of the study, comparative statistics were not performed. All data are presented descriptively in percentages. The initial analysis was conducted to determine any positive or negative sensory shift with repeated applications. More positive shifts were consistently observed across all five domains in the QTZ + SOC study population (Table 2). To determine treatment effect more accurately on sensation with QTZ in patients with no sensation and abnormally low sensation at baseline, two subgroup analyses were conducted, respectively. The proportion of patients treated with QTZ for 30 min + SOC in each subgroup reporting improved sensory perception increasing with repeated applications.

In subgroup one (score of zero at baseline), the percentage of sensory tests increased over repeated applications in all five sensory modalities (Figure 2). In subgroup two (abnormally low sensation at baseline), the shift to normal sensation, was also increased by the 6th application in all five sensory modalities (Figure 3).

**Conclusions:** Improvements in sensory perception were observed with repeated applications of QTZ in patients with DPN. Treatment with high concentration capsaicin ensures sufficient selective binding to transient receptor potential vanilloid 1 (TRPV1)‐expressing nociceptive fibres, resulting in a reversible chemical ablation leading to significant pain relief.^9‐12^ A secondary effect of capsaicin is the release of calcitonin gene‐related peptide (CGRP) in the skin, which is likely the main initiator of neurogenic inflammation, characterized by increased blood flow resulting in edema and erythema.^13,14^ When the pharmacokinetic properties of QTZ were determined, it was hypothesized that the matrix technology in the topical system should ensure the formation of a reservoir of capsaicin in the stratum corneum.^15^ This reservoir may underlie some longer‐term effects of QTZ such as healthier nerve regeneration, improvements in sensory function and nociception. This hypothesis has been supported by recent data showing increased epidermal nerve fibre density (ENFD) at 3 months following an application of QTZ in patients with DPN as compared to baseline ENFD biopsies12. In this study, axon‐reflex vasodilation in the skin was significantly increased between baseline and 3‐month follow‐up in the subset of subjects tested. Additionally, there was a positive correlation between the increase of axon‐reflex vasodilation and ENFD at 3 months post QTZ application. These positive effects on ENFD were time dependent and began to revert to baseline within 6 to 9 months, which should be expected due to the progressive nature of DPN5,12. Given these findings, it is reasonable to assume a long‐term secondary effect of capsaicin on blood flow may result in healthier nerves distally in diabetic patients. Furthermore, the negative shifts observed in this post‐hoc analysis may be the inevitable result of the pathological consequences of diabetes mellitus. The results of this post‐hoc analysis suggest that local vasodilation may result in better neuronal function, and therefore improved sensory perception reported in the PACE trial. More research is warranted to expand on these findings and better understand the benefits of QTZ for patients with DPN.

## 29 IMPLEMENTING A MULTIDISCIPLINARY LIMB SALVAGE TEAM APPROACH TO REDUCE SPLIT‐THICKNESS SKIN GRAFT FAILURE FOR NON‐HEALING LOWER EXTREMITY WOUNDS

### 29.1 **Authors:** James Martinson, MD, MedStar Franklin Square Medical Center; Zoe Haffner, MD, MedStar Georgetown University Hospital; Adah Sayyad, MedStar Georgetown University Hospital; David Martin, MedStar Good Samaritan Hospital; Vinay Gupta, MedStar Franklin Square Medical Center

**Background:** Split‐thickness skin grafting (STSG) is common in wound reconstruction, with much research devoted towards healing. We investigated the influence of a multidisciplinary limb salvage team (MLST) in determining successful and durable take.

**Methods:** Patients undergoing lower‐extremity STSG within our hospital system between 2015 and 2022 were included, where a MLST approach was implemented in 2019. Primary outcomes included successful take (considered at >80%) and failure at any time point (STSG loss to <70% take). Other variable collected included patient demographics and comorbidities, socioeconomic status (defined as having Medicaid insurance or not having insurance), need for revascularization, indication for STSG, and post‐operative wound care. Bivariate analyses were performed with Fisher's Test for Exactness and Mann–Whitney *U* tests, with a priori significance set at 0.05. Bivariate and multivariate logistic regressions were performed to further assess for potential confounders.

**Results:** 128 patients were included, where 75 were treated before MLST implementation and 53 afterwards. Bivariate analyses showed that those treated after MLST were younger (57 years vs. 64 years, *p* = 0.043), were more frequently treated with Negative Pressure Wound Therapy (NPWT) post‐operatively (98% vs. 81%, *p* = 0.004), had professional medical help provided on discharge (57% vs. 17%, p ≤ 0.001), and had lower rates of Peripheral Arterial Disease without any difference in revascularization (42% vs. 61%, *p* = 0.032; 23% vs. 24%, *p* = 1.000). While patients after MLST had an insignificant increase in STSG take (91% vs. 81%, *p* = 0.208), they also had a significantly decreased rate of failure at any time point (15% vs. 37%, *p* = 0.009). Bivariate logistic regression showed no significance in an MLST approach towards successful take, but did demonstrate a decreased odds of failure (OR 0.28, 95% CI 0.10–0.73). However, on multivariate regression, this significance was lost as a lower socioeconomic status and the use of NPWT were found to have higher odds of failure (OR 5.1 (95% CI 2.0–13) and OR 0.12 (95% CI 0.02–0.57), respectively).

**Conclusions:** Our analysis showed that while patients treated with an MLST had lower rates of failure, the patient's socioeconomic status and post‐operative care with NPWT were more influential. Larger, prospective studies are needed to confirm trends lacking significance and implement possible interventions.

## 30 LIMB SALVAGE USING THE REVERSE PERONEUS BREVIS MUSLCE FLAP IN MULTI‐MORBID PATIENTS WITH FOOT & ANKLE SOFT TISSUE DEFECTS

### 30.1 **Authors:** Christopher Bibbo, DO, Sinai Hospital of Baltimore; Suhail Masedah, DPM, University of Cincinnati

**Background:** Soft tissue defects of the F&A remain a challenge in the multi‐morbid patients. The authors present the data of single surgeon experience (CB) of the reverse peroneus brevis (Rev PB) muscle flap in limb salvage for F&A soft tissue coverage in multi‐morbid, high‐risk patients, who otherwise would have undergone a major high level amputation.

**Methods:** Under institutional IRB approval, consecutive multi‐morbid patients wt high‐risk for no other alternative but high‐level amputation, underwent a Rev peroneus breves muscle flap for soft tissue coverage of the foot and ankle.

Patients were followed for complications and success for limb salvage.

**Results:** Patients underwent Doppler examination only prior to execution the Rev PB flap‐ angiography or CT angiogram was not performed.

Of 36 patients enrolled, Males =36%; mean age = 49Y; Mean F/U = 4.6 years. Left = 55%; 72% trauma related; leg = 15%, ankle 59%, foot 26%. 92% patients possessed multiple risk factors for limb loss; median ASA = 3. Infection: 78%.

Flaps harvested on one muscular perforator (22%), two perforators (56%, three perforators (22%). Flap composition: 89% muscle only, 6% flaps either myocutaneous, or myosseous. Skin grafting performed in 94% of patients: 91% split thickness (75% delayed); 9% full thickness (100% immediate). Entire flap survival = 75%; 14% partial flap necrosis; 11% complete flap necrosis. Patients with partial/complete flap loss possessed 4–6 risk factors. The presence of peripheral vascular disease, diabetes or infection alone resulting in partial/complete flap loss was not statistically significant; smoking was a significant single influence on partial/full flap failure (*p* > 0.05, Chi Square). Donor site morbidity: 11% partial incision necrosis. Mean F/U = 4.6 years, 83% limb salvage rate.

**Conclusions:** The data gleaned from our study demonstrates that F&A soft tissue defects in multi‐morbid patients, who are otherwise facing a higher level major amputation, successful limb salvage may be achieved with a Rev PB muscle flap, which should be considered a first line flap prior to free flap.

## 31 *MANAGEMENT OF WOUNDS WITH A COLLAGEN WOUND MATRIX ANTIMICROBIAL BARRIER TO SUPPORT INTRINSIC WOUND HEALING AND PREVENTION OF BIOBURDEN REFORMATION

### 31.1 **Authors:** Justin Avery, PhD, Organogenesis; Kelly Kimmerling, PhD, Organogenesis; Joel Gil, University of Miami; Katie Mowry, PhD, Organogenesis; Stephen Davis, University of Miami

**Background:** Diabetic patients have a 25% incidence rate to develop diabetic foot ulcers (DFU), with non‐healing ulcers accounting for 85% of lower extremity amputations. Biofilm is present in over 90% of chronic wounds inhibiting healing2, and sharp debridement alone may not be sufficient to prevent bioburden and biofilm reformation. Herein, we evaluated a native crosslinked collagen matrix with polyhexamethylene biguanide (PCMP*) as a protective antimicrobial barrier in a biofilm infected porcine wound model.

**Methods:** Deep reticular dermal wounds (4 cm×4 cm×3 mm) on specific pathogen‐free pigs were inoculated with MRSA USA300 and developed a biofilm for 72 h. After biofilm formation, wounds underwent sharp debridement and were covered with PCMP for up to 20 days. At 5, 10, and 15 days, PCMP was removed on some wounds for bioburden level assessment.

**Results:** At all timepoints, PCMP treated wounds had reduced MRSA counts compared to debridement only and resulted in progression of wounds to 90% closure by 2 weeks. Interestingly, removal of PCMP at any point prior to healing resulted in significant resurgence of bioburden (*p* < 0.05).

**Conclusions:** This study highlights the value to applying PCMP through closure to support the intrinsic healing process, reduction of bioburden and prevention of biofilm reformation.

## 32 NO‐INCISION FLOATING DISTAL METATARSAL METAPHYEAL OSTEOTOMY TECHNIQUE: A CASE SERIES

### 32.1 **Authors:** Ersta Ferryanto, DPM, Ascension Saint Joseph Chicago; Haywan Chiu, DPM, Alburquerque Associated Podiatrists

**Background:** Submetetarsal head neuropathic ulcerations are common but highly morbid complications of diabetes. They have a high recurrence rate and are often treated with distal metatarsal metaphyseal osteotomies or floating metatarsal osteotomies. Their efficacy in treating neuropathic diabetic ulcerations compared to nonsurgical approaches is well supported in the literature. The procedure is safe and effective with both open and minimally invasive techniques, but complications do exist. In an effort to further reduce complication rates, we describe a novel no‐incision approach to distal metatarsal metaphyseal osteotomies utilizing a single Kirschner wire. The results of this technique performed in 4 patients were reported after 12 months of follow‐up.

**Methods:** From August 17th, 2021 to October 12th, 2022, 4 patients with neuropathic sub‐metatarsal head ulcerations were treated with percutaneous 0.062” K‐wire DMMO. Our senior primary author was the single‐performing surgeon for our cohort. Before the intervention, all plantar metatarsal head ulcerations were recalcitrant to conservative treatments over 4–12 months including felt pad offloading, and prescription shoes. Ulceration sizes ranged from 0.5 cm × 0.2 cm × 0.1 cm full thickness to 1.3 cm × 1.3 cm × 0.1 cm. Healing was defined as full epithelization of the ulcer.

**Results:** All 4 patients healed their plantar metatarsal ulcerations within 16 weeks of the procedure. No transfer ulcerations were noted at the final follow‐up period for each patient. There were no surgical site complications at the final follow‐up. The mean follow‐up period was between 9 and 23 months with an average time of 15.25 months. All patients' ulcers were healed between 3 and 16 weeks’ time with an average time of 8.5 weeks. No complications were noted for all patients at the most recent follow‐up time.

**Conclusions:** In our cohort, all patients healed their ulcers within 16 weeks without recurrence or complication at the final follow‐up. No infections, transfer lesions, or nonunions were noted. We demonstrate successful results with our modification of a popular procedure. Although we found high success in our small cohort, more research is required to determine its reproducibility and efficacy. We believe our non‐incision metatarsal head osteotomy shows promising initial outcomes for the treatment of plantar lesser metatarsal head ulcerations.

## 33 *PRECLINICAL, EASE OF USE, AND INITIAL CLINICAL ASSESSMENTS OF A LONGER‐WEAR, PEEL AND PLACE NEGATIVE PRESSURE WOUND THERAPY (NPWT) DRESSING

### 33.1 **Authors:** Kristine Kieswetter, PhD, Solventum; Diwi Allen, MS, Solventum; Samantha Mann, Solventum; Sara Pike, Solventum; Marisa Schmidt, Solventum

**Background:** Reticulated open cell foam (ROCF)^ is well‐established for use with NPWT*, but tissue ingrowth may occur if left in place >3 days. A novel peel and place dressing† was created to address this challenge.

**Methods:** Peel and place dressing or ROCF plus interface layer (IFL) were applied with weekly dressing changes to full‐thickness (13‐day study) and deep muscle wounds (35‐day study) on swine at −125 mmHg NPWT. Histopathology was performed. Animal work was approved by relevant Institutional Animal Care and Use Committee (IACUC) and complied with applicable national/local regulations. Also, usability studies were conducted to determine if participants could successfully complete dressing application and sealing. Lastly, initial clinical evaluations of the peel and place dressing were performed on complex geometries.

**Results:** Preclinical assessments indicated greater granulation (*p* < 0.0001) with comparable re‐epithelialization versus ROCF+IFL/IFL (13‐day study). Tissue ingrowth was limited. The deep muscle wounds proceeded to full closure (35‐day study). Additionally, participants found the dressing easier and faster (2.6×) to use while successfully completing dressing application and sealing. Furthermore, the dressing was successfully used on a diabetic foot wound.

**Conclusions:** At day 13, greater tissue regeneration was promoted and tissue ingrowth mitigated in swine. Deep muscle wounds closure was successfully facilitated with only 4 dressing changes (35‐day study). These findings support the suitability of the peel and place dressing for 7‐day wear. Plus, users applied the peel and place dressing more quickly and easily. Additionally, early feedback on clinical use of the peel and place dressing in highly contoured areas was positive.

^3M™ V.A.C.® Granufoam™ Dressing; *3M™ V.A.C.® Therapy; †3MV.A.C.® Peel and Place Dressing (3M, San Antonio, TX, USA).

## 34 QUALITATIVE AND QUANTITATIVE PERCEIVED BARRIERS TO LOWER EXTREMITY WOUND CARE AMONG UNDERSERVED POPULATIONS WITH DIABETES IN URBAN AREAS

### 34.1 **Authors:** Trinh Ho, Temple University School of Medicine; Abigail Anderson, Temple University School of Podiatric Medicine; Jessica Carrillo, Temple University School of Podiatric Medicine; Courteney Asase, Temple University School of Podiatric Medicine; Andrew Meyer, DPM, Temple University School of Podiatric Medicine

**Background:** Diabetes‐related foot complications, particularly Diabetic Foot Ulcers (DFUs), are a major cause of morbidity and mortality among individuals with diabetes. The objective is to characterize patient‐perceived barriers among patients with DFUs in an urban population. By identifying these barriers, we hope that targeted interventions improving wound care management might be developed.

**Methods:** A qualitative and quantitative study was conducted by semi‐structured interview with patients with DFUs at the Temple Foot and Ankle Institute. Data were analysed through content analysis by developing main themes and categories.

**Results:** After conducting interviews with 24 patients, three primary themes with associated subcategories were discerned: Theme 1—Cultural and Belief Barriers, comprised of two categories: (i) an exaggerated belief in the healthcare provider's paramount importance for wound healing, and (ii) cultural wound care practices. Theme 2—Accessibility and Financial Barriers encompassed three categories: (i) healthcare facility availability and transportation, (ii) a gender disparity that females are twice as likely to receive help with foot care at home, and (iii) financial responsibilities and appointment scheduling. Theme 3—Patient Education Barriers, comprising three categories: (i) identification and treatment of wounds, (ii) management of diabetes and overall health, and (iii) awareness of risk factors associated with diabetic wounds and amputations.

**Conclusions:** Patients with DFUs expressed encountering substantial external barriers, which upon further investigations, may foster misconceptions that downplay the significance of individual patient responsibility and adherence. Additional barriers include gender discrepancies in home‐based care and variations in the extent and accuracy of their knowledge regarding DFUs.

## 35 *REFINING AND EXPANDING FREE TISSUE TRANSFER FOR THE COMORBID, CHRONIC WOUND POPULATION: ANALYSING MULTIPLEX SURGICAL CARE IN 300 LOWER EXTREMITY FREE FLAPS

### 35.1 **Authors:** Nisha Gupta, MedStar Georgetown University Hospital; Karen Li, Georgetown University School of Medicine; Christian Lava, MS, MedStar Georgetown University Hospital; Christopher Attinger, MD, MedStar Georgetown University Hospital; Karen Evans, MD, MedStar Georgetown University Hospital

**Background:** Free tissue transfer (FTT) for lower extremity (LE) salvage in the chronic wound, co‐morbid population requires a multidisciplinary team invested in delivering multiplex surgical care. It is critical to analyse overall outcomes in this highly complex and high healthcare expenditure population.

**Methods:** A retrospective review of 300 LE FTTs by a single surgeon was performed, comparing the first 200 (July 2011–February 2020) to the most recent 100 (February 2020–January 2023) patients. Demographics, comorbidities, preoperative management, intraoperative details, and outcomes were collected.

**Results:** In the group of most recent 100 FTTs, patients had a higher prevalence of diabetes (67.0% vs. 48.5%, *p* = 0.002) and peripheral vascular disease (56.0% vs. 24.5%, p&lt;0.000). Preoperative imaging showed higher rates of venous reflux (81.9% vs. 67.8%; *p* = 0.034) and venous thrombosis (25.5% vs. 10.5%; *p* = 0.003). They also received more preoperative endovascular interventions (23.0% vs. 16.5%, *p* = 0.039). Intraoperatively, this group had a greater proportion of calcified vessels (43.0% vs. 20.5%; *p*&lt;0.001). Rates of partial flap necrosis were higher for this group (7% vs. 3%; *p* = 0.012). However, no other significant differences were found in rates of early reoperation, hematoma, or infection. The most recent 100 FTT had greater flap success (98% vs. 95.5% vs.; *p* = 0.347) and shorter median time to ambulation (2.23 vs. 4.37 months; *p* = 0.000).

**Conclusions:** The multidisciplinary care model is critical for success in this complex patient population. With increasing surgical experience, case volume and repetition, the multiplex care team can offer higher success rates and enhanced outcomes while offering FTT to an overall sicker population.

## 36 RISK FACTORS FOR EVENTUAL LIMB LOSS FOLLOWING FREE TISSUE TRANSFER IN PATIENTS WITH PERIPHERAL VASCULAR DISEASE—LONG TERM FOLLOW UP

### 36.1 **Authors:** Sabrina DeLeonibus, MS, Georgetown University School of Medicine; Karen Li, Georgetown University School of Medicine; Christian Lava, MS, MedStar Georgetown University School of Medicine; Christopher Attinger, MD, MedStar Georgetown University School of Medicine; Karen Evans, MD, MedStar Georgetown University School of Medicine

**Background:** The prevalence of peripheral vascular disease (PVD) and other comorbidities in patients undergoing (LE) limb salvage using free tissue transfer (FTT) remain as risk factors for chronic limb ischemia and amputation. The aim of this study is to determine the risk factors for limb loss in patients with PVD receiving LE FTT.

**Methods:** LE FTTs performed by a single surgeon from July 2011 to January 2023 were reviewed. Preoperative angiograms were reviewed for evidence of arterial abnormalities. Univariate analyses were performed to determine significant covariates to the primary outcome, which was progression to ipsilateral amputation.

**Results:** Among 300 patients who received LE FTT, there were 130 patients with evidence of diseased vasculature (PVD). The PVD group had significantly higher rates of comorbidities including diabetes (66.2% vs. 45.9%, *p* = 0.000), neuropathy (52.3% vs. 36.1%, *p* = 0.005), chronic kidney disease (CKD) (22.3% vs. 10.6%, *p* = 0.007), and congestive heart failure (8.5% vs. 2.9%, *p* = 0.035). On postoperative complications, the PVD cohort had significantly increased rates of dehiscence (20.8% vs. 11.8%, *p* = 0.033) and infection (19.2% vs. 10.0%, *p* = 0.022). The overall amputation rate was not significant between two groups (PVD:15.4% vs. No PVD: 10.6%, *p* = 0.216).

**Conclusions:** Patients with a complex history of comorbidities appeared to be at a much higher risk for eventual amputation. Close follow‐up in these patients focused on medical management and infection control are needed to achieve long‐term limb salvage.

## 37 RISK FACTORS FOR LIMB LOSS FOLLOWING FREE TISSUE TRANSFER IN THE COMORBID POPULATION: LONG‐TERM OUTCOMES IN 300 PATIENTS

### 37.1 **Authors:** Karen Li, Georgetown University School of Medicine; Christian Lava, MS, MedStar Georgetown University School of Medicine; Woori Lee, Georgetown University School of Medicine; John Steinberg, DPM, MedStar Georgetown University School of Medicine; Karen Evans, MD, MedStar Georgetown University School of Medicine

**Background:** Diabetes and PAD are top causes of non‐traumatic amputation in the US. The debate on optimal criteria for limb salvage or amputation persists, especially in complex cases. Lower extremity (LE) free tissue transfer (FTT) is an efficacious reconstructive tool aiding successful limb salvage. Despite efforts, comorbid patients remain at risk of amputation. This study aims to identify amputation risk factors following microsurgical FTT in the nontraumatic chronic wound population.

**Methods:** A single‐institution, retrospective review of LE FTT patients (June 2011–December 2023) collected demographics, medical history, Charlson Comorbidity Index (CCI), preoperative laboratory testing, preoperative angiogram details, microsurgical details, complications, and long‐term outcomes. Patients who progressed to amputation (AMP) were compared to patients with successful limb salvage (LS). Variables conferring future amputation risk were examined using univariate logistic regression.

**Results:** 300 LE FTT patients had a 12.7% (*n* = 38/300) amputation rate at a median of 168 (IQR:220) days. Of the 38 amputations, 63% were attributed to infection, 13% to flap failure, 11% to critical limb ischemia, 3% to hematoma, and 11% to other causes.

In comparison to the LS cohort, patients in the AMP cohort had significantly higher CCI (4 vs. 5, *p* = 0.0375), Longer length of stay (25 vs. 34.5 days, *p* = 0.0001), and longer postoperative length of stay (14 vs. 17 days, p = 0.001). The pre‐operative vascular status revealed no significant differences in vessel runoffs between the AMP and LS cohorts and rates of pre‐LE FTT vascular interventions were comparable (AMP = 23.7% vs. LS = 17.9%, *p* = 0.396). Patients in the AMP cohort had significantly higher rates of wounds in the hindfoot (36.8% vs. 19.5%, *p* = 0.015) and plantar foot (34.2% vs. 17.2%, *p* = 0.013). Postoperative complications were markedly higher for the AMP group (57.9% vs. 24.1%, *p* = 0.000), including delayed complications like dehiscence (OR: 2.56, *p* = 0.019), infection (OR: 4.875, p = 0.0000), and recurrence of osteomyelitis (OR: 8.60, p = 0.000) prior to amputation.

On univariate analysis, risk factors for amputation included diabetes (OR: 3.582, CI:1.582–8.106), neuropathy (OR: 3.649, CI:1.728–7.703), CKD (OR: 1.867, CI:1.276–2.731), higher CCI (OR: 1.157, CI: 1.003–1.334), ESRD (OR: 5.271, CI:1.761–15.779), hindfoot defects (OR: 2.413, CI:1.167–4.991), plantar foot defects (OR: 2.507, CI: 1.193–5.272), history of Charcot arthropathy (OR: 4.207, CI: 1.731–10.223), and elevated a1c levels (OR: 1.203, CI:1.061–1.365). On multivariate logistic regression, history of Charcot arthropathy remained a significant risk predictor of amputation (OR:3.2, CI:1.2–8.3).

**Conclusions:** Multiple risk factors for amputation following LE FTT patients with non‐traumatic chronic wounds were identified. This study informs optimal treatment strategies, potentially enhancing overall management in complex limb salvage decisions.

## 38 SAFETY AND EFFICACY OF CONTINUOUS DIFFUSION OF OXYGEN FOR SURGICALLY CLOSED WOUNDS IN PATIENTS WITH DIABETES AND PAD—A PILOT RANDOMIZED CONTROLLED TRIAL STUDY

### 38.1 **Authors:** Miguel Bargas Ochoa, MD, Baylor College of Medicine; Alejandro Zulbaran Rojas, MD, Baylor College of Medicine; Bernardo Martinez Leal, MD, Baylor College of Medicine; Tyler L. Coye, MD, Baylor College of Medicine; Bijan Najafi, PhD, Baylor College of Medicine

**Background:** Patients with peripheral arterial disease (PAD) and diabetes mellitus (DM) developing foot ischemia encompass around 24% conversion rate to minor amputations.^1^ Such procedures may conclude with a primary closure that can fail due to necrosis infection, and/or wound dehiscence.^2^ We examined the effect of continuous diffusion of oxygen (CDO) to aid surgically closed wounds within this population.

**Methods:** A 4‐week RCT in patients with PAD+DM undergoing minor amputations following primary closure was performed. Patients were randomized depending on post‐operative treatment either to standard of care (SOC) or SOC + CDO, delivered via a cannula connected to an oxygen device. Safety outcomes included proportion of wound complications (dehiscence, necrosis, infection) and hospital readmissions/re‐operations at 4‐week. Furthermore, an 8‐week observational period evaluated efficacy outcomes via survival analysis, including time‐to‐wound healing and hospital readmissions.

**Results:** Sixteen patients were included (SOC, *n* = 10; SOC + CDO, *n* = 6). Baseline clinical characteristics were not significantly different between groups. Safety outcomes revealed lower rates of wound complications (16% vs. 30%, *p* = 0.5) and hospital readmissions (0% vs. 20%, *p* = 0.24) in the SOC + CDO group compared to the SOC group. Efficacy outcomes showed a faster time to heal (61.2 ± 14.1 days vs. 73.7 ± 8.5 days, log rank: *p* = 0.46) and longer time for hospital readmissions (89 ± 0.8 days vs. 73.3 ± 9.7 days, log rank: *p* = 0.3) in the SOC + CDO group compared to the SOC group. However, these results were not statistically significant.

**Conclusions:** Postoperative SOC + CDO showed a trend to reducing wound complications and fasten time to heal in PAD+DM patients undergoing minor amputations with primary closure. A larger sample size is warranted to validate these observations.

## 39 SINGLE DOSE RADIATION AS AN ADJUNCTIVE THERAPY WITH SURGICAL RESECTION FOR PATIENTS WITH SYMPTOMATIC HETEROTROPHIC OSSIFICATION POST AMPUTATION; A CASE REPORT

### 39.1 **Authors:** Aliza Lee, DPM, SVAMC; Benjamin Klopfenstein, DPM, SVAMC; Venita Cucurella Smith, DPM, SVAMC; David Asher, MD, Blue Ridge Cancer Care

**Background:** 80% of the 120 000 nontraumatic amputations preformed yearly are contributed to diabetes. Diabetes is more prevalent among US veterans, who make up 9% of the civilian US population, than among the general population and affects nearly 25% of all Veteran Affairs (VA) patients. Formation of extra skeletal bone in muscle and soft tissues is a common complication of trauma, surgery, and other local or systemic insults. Heterotopic ossifications maybe a finding after foot amputation with up to 23% occurrence. Sequela from amputation can predispose excessive pressure on the weightbearing surface of the foot and subsequent wound formation. In those instances, perioperative use of pharmacologic and/or radiation prophylaxis may be warranted. Low‐dose radiation has been studied both as a prophylactic modality for primary prevention in high‐risk patients or as a prophylactic modality as secondary prevention together with surgical excision. Prophylactic doses and are given either 24 h preoperatively or up to 72 h postoperatively. Presented is a case report of a 55 y/o uncontrolled diabetic male with neuropathy that underwent partial 1st ray amputation after diabetic foot infection, eventual trans‐metatarsal amputation with tendo‐achillies lengthening and had progressive bone formation that lead to ulceration with pain and the need for surgical intervention for symptomatic relief and restoration of foot function.

**Methods:** The morning of surgery, a single fraction of external beam radiation therapy was delivered to a dose of 700 cGy using two opposed 6 MV photon fields with coverage of the left foot. Later that day at another location, the plantar diabetic foot ulceration was excised in a fish mouth manner encompassing the ulceration. Next exposure was carried down to bone. Tissue dissection was performed creating a large dorsal and plantar skin flap over the remaining metatarsal bones. During the dissection metatarsal shafts 1 through 4 were noted to be with irregularity in that they were with hypertrophic and with morphological changes. After adequate amount of bone was exposed the metatarsal shafts were resected with a sagittal saw to clean and even margins. The heterotrophic ossification of the first ray was examined on the back table and was noted to be with irregular trabecular bone pattern and density. The remaining 1st metatarsal was removed. Bone block with ulceration was sent to pathology for confirmation and to rule out malignant or osteolytic process. Incisional vac was placed and patient sent home.

**Results:** The pathology reports describe a tadpole‐shaped fragment 11 × 4 × 2.5 cm (LxWxD). The head of the fragment consists of a circular 5 × 4.5 cm(LxW) portion of white skin with a 3.8 cm circumferential diameter ulcer with inflammation and fibrosis with subjacent bone displaying marrow fibrosis and osteo‐neogenesis. The incision slowly epithelized over the next 12 weeks. Repeat x‐ray at 12 weeks demonstrated minimal radiographic asymptomatic bone growth to the 5th metatarsal bone, 2–4 metatarsals with no growth, and patient reported resolution in foot pain.

**Conclusions:** Heterotrophic ossification of amputation sites in an already high‐risk patient population poses a threat to limb salvage efforts. Minimizing the risk of re‐ulceration/re‐amputation in neuropathic diabetic patient population is of importance. Single‐dose radiation therapy has been documented to help prevent bone formation and may act as an effective prophylaxis adjunct to surgical resection.

## 40 SOCIOECONOMIC AND RACIAL DISPARITIES IN LIMB SALVAGE FOR PATIENTS WITH DIABETIC LOWER EXTREMITY WOUNDS

### 40.1 **Authors:** Pooja Humar, University of Pittsburgh Medical Center; Elizabeth Moroni, MD, University of Pittsburgh Medical Center; Casey Zhang, University of Pittsburgh Medical Center; J. Peter Rubin, MD, MBA, University of Pittsburgh Medical Center; Brodie Parent, MD, University of Pittsburgh Medical Center

**Background:** Diabetic wounds are a leading cause of lower extremity (LE) amputations and disparities in care influence the likelihood of undergoing salvage procedures versus amputation. The Area Deprivation Index (ADI) and Social Vulnerability Index (SVI) capture neighbourhood factors associated with health outcomes. This study assesses the influence of ADI, SVI, and distance to a wound center on healing and limb loss in patients with diabetic LE wounds.

**Methods:** A retrospective cohort study (2015–2022) of 17 wound care clinics was used to identify patients with ⎕1 LE wound. Regression analysis was performed to determine which covariates predicted undergoing lower extremity surgical procedure (LESP) and/or amputation.

**Results:** Patients living in lower ADI neighbourhoods, indicating improved conditions across income, education, employment, and housing, were more likely to have more healed wounds (*p* = 0.03). Living >50 miles from a wound care center was associated with a greater number of LESP (OR = 1.18, 95% CI 1.01–1.34, *p* = 0.04), including emergency department or inpatient visits requiring operative debridement. White patients were half as likely to undergo additional LESP (OR = 0.9033, 95% CI 0.85–0.96, *p* = 0.0003). However, they were more likely to have outpatient encounters for routine wound care compared to racial/ethnic minorities (OR = 1.01, 95% CI 1.00–1.02, *p* = 0.01). Patients living in areas with higher‐than‐average unemployment were more likely to receive an amputation (HR = 14.45, 95% CI 12.28–16.63, *p*‐value = 0.016).

**Conclusions:** In patients with diabetic LE wounds, racial/ethnic minorities and lower socioeconomic status were associated with more LESP and amputations. This data can target factors such as identifying implicit‐bias, education, and healthcare access to incorporate into limb salvage programs.

## 41 SPLIT‐THICKNESS SKIN GRAFT OUTCOMES IN NONTRAUMATIC LOWER EXTREMITY WOUNDS: DOES LOCATION MATTER?

### 41.1 **Authors:** Rachel Rohrich, MedStar Georgetown University Hospital; Karen Li, Georgetown University School of Medicine; Christian Lava, MS, MedStar Georgetown University Hospital; Henry Stanton, Georgetown University School of Medicine; Christopher Attinger, MD, MedStar Georgetown University Hospital

**Background:** The use of a split‐thickness skin graft (STSG) is a mainstay for managing chronic, nontraumatic lower extremity (LE) wounds. However, the biomechanics of LE anatomy introduces shearing forces and natural pressure points that can lead to STSG failure. Such challenges may deter surgeons from considering STSG in regions such as the heel and ankle when such efforts could lead to successful limb salvage. This study aims to determine if wound location and surface impacts STSG outcomes.

**Methods:** A retrospective review of patients who underwent STSG from December 2014 to December 2022 was conducted. Wounds pre‐treated with synthetic dermal matrix (SDS) prior to STSG placement were excluded. Patient demographics, wound characteristics, and post‐operative outcomes were collected. Wound location was classified into seven categories: forefoot, midfoot, hindfoot, transmetatarsal amputation (TMA) site, ankle, leg, and knee. Foot wounds were further classified as plantar or dorsal. Graft failure, defined as complete necrosis or removal of the STSG, was compared among groups. An additional sub‐analysis of plantar wounds treated with and without SDS was performed.

**Results:** A total of 168 patients with 245 wounds underwent STSG during the study period. Overall, the cohort was 61.3% male with a median age of 61.9 (IQR: 15.1) years and BMI of 28.5 (IQR: 8.9) kg/m^2^. The median Charlson Comorbidity Index (CCI) score was 4 (IQR: 3), reflecting prevalent rates of diabetes mellitus (DM) (57.1%), chronic kidney disease (CKD) (22.6%) and peripheral artery disease (PAD) (36.9%). Median wound size was 29 (IQR: 71) cm^2^. Wounds were located on the forefoot (*n* = 48/245, 19.6%), midfoot (*n* = 20/245, 8.2%), hindfoot (*n* = 36/245, 14.8%), ankle (*n* = 45/245, 18.4%), lower leg (*n* = 78/245, 31.8%), knee (*n* = 15/245, 6.1%), and TMA (*n* = 5/245. 2.0%). Overall, rate of graft failure was 18.0% (*n* = 44/245), with no differences between location groups (*p* = 0.601). In the foot, wounds on plantar surfaces exhibited significantly higher rates of graft failure compared to dorsal defects (*n* = 11/37, 29.7% vs. *n* = 8/70, 11.4%, *p* = 0.018). Furthermore, in a univariate regression, plantar foot wounds were associated with a 3.3‐fold increase in the odds of graft failure (OR: 3.3, CI: [1.1, 9.1], *p* = 0.022). Subanalysis of plantar defects treated with and without SDS prior to STSG demonstrated that pre‐treatment with SDS decreases graft failure rates significantly by 23.6% (7.1% vs. 30.8%, *p* = 0.019).

**Conclusions:** Our results suggest that STSG is a viable method for LE wound coverage across multiple locations. However, wounds on plantar surfaces are more susceptible to graft failure. In these cases, the use of SDS prior to STSG may increase chances of graft success. Our institution's rigorous approach to postoperative immobilization and ambulation restrictions may have contributed to the overall success of STSG in our highly comorbid cohort. Emphasizing off‐loading protocols is critical to improve graft outcomes in high‐risk areas in a comorbid patient population.

## 42 *THE ECONOMIC IMPACT OF REDUCING HOSPITALIZATIONS AND AMPUTATIONS IN PATIENTS WITH DIABETIC FOOT ULCERS

### 42.1 **Author:** Matthew Garoufalis, DPM, PFCS, PC, Professional Foot Care Specialists

**Background:** The purpose of this real‐word evidence (RWE) cohort study was to evaluate whether utilization of Cyclical Pressurized Topical Oxygen (TWO2) therapy had any impact on the incidence of Diabetic Foot Ulcer related hospitalizations and amputations over a 12‐month period in patients with hard to heal Diabetic foot Ulcers (DFU).

**Methods:** An IRB approved retrospective review of deidentified DFU patient medical records was conducted at 2 US Veterans Affairs Medical Centers in Illinois and Virginia, which included demographic information on wound characteristics, clinical characteristics, neuropathy, cardiovascular disease (CVD), peripheral vascular disease (PVD), pain levels and kidney disease etc.

**Results:** Within the unmatched cohorts of the 202 DFU patients (91 TWO2, 111 NO TWO2) there was an 88% reduction in hospitalizations and a 71% reduction in amputations over a 12‐month period for the patients treated with TWO2 therapy compared to those that did not receive TWO2 therapy. Propensity score matching was conducted showing similar outcomes and a regression analysis as preformed along with a budget impact model which demonstrated significant cost saving to the payor system.

**Conclusions:** This large RWE study demonstrates the TWO2 therapy positively impacts patients quality of life and the costs associated with treating their DFU by forcing them into remission, resulting in significantly reduced rates of hospitalizations, amputations and cost outlay.

## 43 *THE IMPACT OF A PROPHYLACTIC TENDO‐ACHILLES LENGTHENING WITH TRANSMETATARSAL AMPUTATION: A MATCHED COHORT STUDY

### 43.1 **Authors:** Tiffanie Liu, DPM, MS, MedStar Georgetown University Hospital; Jessica Arneson, DPM: John Steinberg, DPM, MedStar Georgetown University Hospital; Christopher Attinger, MD, MedStar Georgetown University Hospital; Tammer Elmarsafi, DPM, Vascular Surgery Associates

**Background:** A TMA is a viable limb salvage option to preserve length and function, allowing for ambulation without a prosthetic and with less energy expenditure when compared to higher level amputations (1). A majority of TMAs occur in the diabetic population. In 2020, 160 000 diabetic patients were hospitalized for amputations (2).

Amputation at the level of the metatarsal shafts transects the long extensors, allowing the posterior muscle group to gain mechanical advantage. This often results in an equinus deformity (3). Equinus is described as limited ankle dorsiflexion (<10°) (4). With equinus, patients are prone to touch the ground with the forefoot during attempted heel strike. Garbalosa et al. confirmed this when he found a significantly greater mean peak plantar pressure in the post‐TMA foot than in the contralateral foot of the same patients (5). This issue is compounded in diabetics due to an abnormal collagenous organization and non‐enzymatic glycosylation in diabetic Achilles' tendons (6). Batista et al. used ultrasound on Achilles tendons, finding abnormalities in structural orientation and noted calcifications in asymptomatic diabetics (7). Lavery et al. noted that the prevalence of equinus was 10.3% in 1666 patients diagnosed with diabetes. They found that patients with equinus had increased peak plantar pressures and had been diabetic for a significantly longer time (8).

A TAL is considered a simple adjunctive procedure often performed with TMAs to reduce forefoot pressures, therefore decreasing the risk of future ulceration (9, 10). But a TAL is not a procedure without any risk. The most well‐known complication is overlengthening—the amount of lengthening achieved is not easy to control and there is risk of rupture. Some physicians are less likely to perform a concomitant TAL with TMA since overlengthening can result in a calcaneal gait, progressing into heel ulcerations, especially in patients with peripheral neuropathy since calcaneal osteomyelitis increases the chances of a major amputation 35%–52% (14, 15).

The primary aim of this study is to create a risk‐adjusted matched cohort to evaluate if there is an increased association of heel ulcerations and major amputation after a TMA with a TAL compared to TMA alone. The secondary aim is to see if there is a decrease in forefoot ulceration after TMA with TAL. The third aim is to investigate postoperative outcomes, specifically ambulation status.

**Methods:** After IRB approval, data was collected for TMAs performed by 5 surgeons from 2010 to 2015 at a single institution. All patients 18 years of age or older who had undergone TMA were enrolled and analysed retrospectively via chart review. The study group was composed of patients who had also undergone TAL at the time of final closure. All TALs were performed using the percutaneous triple hemi‐section technique, with 3 cuts in the tendon at 3, 6, and 9 cm from tendon insertion into the calcaneus (Figure 1).

The study group (TMA + TAL) was matched 1:1 for age, diabetes, peripheral vascular disease, smoking status, and chronic kidney disease with a control group (TMA only). Patients requiring a multi‐staged approach for infection control or vascular optimization as well as single stage TMAs were included. A minimum of 30 days follow‐up data was required. When identifying post‐operative forefoot ulcerations, only new forefoot ulcerations were included while incisional dehiscence, ischemia, or infection were excluded. When identifying post‐operative heel ulcerations, only heel ulcerations with documented calcaneal gait were included while decubitus ulcers were excluded. During the follow‐up period, the need for major lower extremity amputation and time in days since final closure of initial TMA was recorded. Major lower extremity amputation was defined as BKA, through‐knee amputation, or AKA. All patients were ambulatory before TMA was performed. Post‐operative ambulatory outcomes were measured, including ambulation unassisted or assisted with a device (cane, walker, and prosthetic).

The demographic and clinical characteristics of patients undergoing TMA in conjunction with TAL (study group) and patients who received an isolated TMA (control group) were described using mean, median, mode, frequencies, and percentages for categorical variables. Chi‐square and Fishers exact test (when cells had counts less than 5) were used when appropriate to compare proportions of the variables. Propensity score matching based on age, diabetes, peripheral vascular disease, smoking status, and renal disease were used to obtain a matched 1:1 risk adjusted cohort for the cases. Bivariate analysis was performed. Multivariate analysis was not performed as no statistically significant factors were obtained by the bivariate analysis. Statistical significance was defined as *p* < 0.05.

**Results:** Data was collected for 228 transmetatarsal amputations that were performed by five surgeons from 2010 through 2015 at a single surgical institution. After application of the inclusion and exclusion criteria, a total of 55 patients (69 feet) including a TAL were identified while a total of 37 patients (39 feet) without a TAL were identified. Two patients lacked an identifiable matched control, yielding 37 matched controls for 37 feet. As previously mentioned, age, diabetes, peripheral vascular disease, smoking status, and renal disease were used to obtain a matched risk‐adjusted cohort between the study and control groups. The average length of follow‐up was 30 months.

In the study group (*n* = 37), the average age was 61.9 + 10.88 years while the average age in the control group (*n* = 37) was 61.9 + 10.21 years. The study and control groups each contained 34 patients with diabetes and 28 patients with peripheral vascular disease. The average haemoglobin a1c in the study group measured 7.73% and 7.55% in the control group. The subject population contained 21 smokers and 22 patients with chronic kidney disease in each group (Table 1).

After intervention, 10 patients (29.4%) in the TAL study group went on to develop a heel ulceration while 6 patients (17.6%) in the control group developed a heel ulceration (*p* = 0.2587). In the study group, 8 patients (23.5%) developed a forefoot ulceration while 7 patients (20.6%) in the control group developed a forefoot ulceration (*p* = 0.7725). Of the 37 patients who received a TMA with TAL, 6 (17.6%) went on to major lower extremity amputation compared to 7 (20.6%) of those who did not receive concomitant TAL (*p* = 0.76). Finally, 32 patients (94.1%) in the study group and 30 patients (88.2%) in the control group were ambulatory after surgery (*p* = 0.5282) (Table 2). As mentioned above, multivariate analysis was not performed as no statistically significant factors were obtained by the bivariate analysis.

**Conclusions:** Tendo‐achilles lengthening is a simple and effective procedure that can be safely done at the same time as the transmetatarsal amputation in hopes of correcting equinus and preventing future forefoot ulcerations. With our data, we ultimately conclude that performing a TAL with a TMA does not increase the risk of heel ulcerations or MLEA (in comparison to no TAL). Performing the TAL may not be a permanent solution to equinus correction, but it may delay the development of forefoot and heel wounds. Given the high rate of failure after a TMA in general (due to a multitude of comorbidities), consideration of adjunctive soft tissue procedures (tendon lengthening, tendon transfers, etc) is an important factor in providing the greatest chance of avoiding further breakdown post operatively. The benchmark for a successful TMA is preventing further limb loss while giving the patient a functional limb to stand and walk on. Proper preoperative evaluation and postoperative risk consideration should always be performed. Regardless of what procedures are performed, we have to remember that this patient population has many comorbidities, making them at high risk for complications and further amputations. Close monitoring and continued follow‐up are therefore necessary.

## 44 THE USE OF DERMAL REGENERATION MATRIX FOR SHORT AND LONG‐TERM LIMB SALVAGE: A COMPARATIVE STUDY OF 402 WOUNDS

### 44.1 **Authors:** Rachel Rohrich, MedStar Georgetown University Hospital; Karen Li, Georgetown University School of Medicine; Christian Lava, MS, MedStar Georgetown University Hospital; Danny Chamaa, MS, Georgetown University School of Medicine; Christopher Attinger, MD, MedStar Georgetown University Hospital

**Background:** A split‐thickness skin graft used alone over exposed tendon, bone, or hardware often fails due to its fragility and susceptibility to shear forces and breakdown. In these cases, the use of dermal regeneration matrix (DRM) prior to STSG is indicated to support granulation formation. While the utility of such products are widely recognized in the wound healing community, there is a lack of comparative studies investigating DRM's efficacy. Our study compares short‐ and long‐term outcomes of DRM use in STSG procedures of the LE.

**Methods:** We reviewed 402 chronic LE wounds in 271 patients who underwent STSG from 2014 to 2022. Outcomes were compared between wounds that received DRM prior to STSG (“DRM”) and those that did not (“Non‐DRM”). Wounds were defined as deep if they had exposed fascia, muscle, tendon, or bone on STSG date. Major amputation was defined as below‐knee or above‐knee amputation. Primary outcomes were STSG loss and amputation.

**Results:** Overall, 175 (43.5%) wounds received DRM and 227 (56.5%) did not. Patients had a median age of 64 years (IQR: 20) and BMI of 28.7 (IQR: 8.7) kg/m^2^. Major comorbidities were diabetes mellitus (DM) (54.2%), peripheral vascular disease (PVD) (38.0%), and chronic kidney disease (CKD) (22.9%), with no significant differences between groups. On STSG date, median wound size was similar between groups (30 cm^2^, IQR: 62), but Non‐DRM wounds demonstrated a higher proportion of deep wounds (36.1% vs. 26.3%, *p* = 0.036). Postoperatively, the DRM group demonstrated significantly less graft loss (16.6% vs. 31.3%, *p* = 0.001) and graft failure (5.1% vs. 19.8%, *p* < 0.001). In a multivariate logistic regression accounting for CKD, DM, PVD, BMI, wound depth and size, and smoking history, DRM was independently associated with an 80% reduction in STSG failure (OR: 0.2, CI: (0.1, 0.4), *p* < 0.001). In the long‐term, we observed similar postoperative amputation rates between groups (Non‐DRM: 18.1% vs. DRM: 13.1%, *p* = 0.181). Both groups displayed similar times to major amputation (552 vs. 581 days, *p* = 0.929), but the DRM group trended a longer median time to minor amputation (1119.5 vs. 548.4, *p* = 0.516).

**Conclusions:** Wounds treated with DRM were more successful in the short‐term, with significantly lower graft loss and failure rates. However, long‐term amputation rates did not show DRM to have the same benefit. Our study suggests that the use of DRM may prolong the time to minor amputations, and that it may be beneficial in a certain risk‐stratified patient in limb salvage. Overall, our results suggest the benefit of DRM as a temporizing measure to enhance STSG take; however, in the long run, DRM use may not mitigate eventual amputation for all chronic LE wound patients.

## 45 THE UTILITY OF NEGATIVE PREOPERATIVE CULTURES IN LOWER EXTREMITY SPLIT‐THICKNESS SKIN GRAFT OUTCOMES

### 45.1 **Authors:** Sami Alahmadi, MS, Georgetown University School of Medicine: Rachel Rohrich, MedStar Georgetown University Hospital; Karen Li, Georgetown University School of Medicine; Christian Lava, MS, MedStar Georgetown University Hospital; Christopher Attinger, MD, MedStar Georgetown University Hospital

**Background:** Serial debridement to achieve a negative culture has become a standard protocol before closure of chronic, lower extremity (LE) wounds. However, in this population, achieving sterility is often not feasible. The current study aims to evaluate the impact of the qualitative debridement cultures obtained immediately before STSG placement on STSG outcomes.

**Methods:** We performed a retrospective review of all patients receiving an STSG for chronic LE wounds from December 2014 to December 2022. Patient demographics, wound characteristics, and post‐operative outcomes were collected. Microbiological data included pathogen type and bacterial load. Wounds that had a preoperative positive culture (PC) on the day of STSG were compared to those that had a negative culture (NC). Primary outcomes were STSG failure, defined as complete necrosis or removal of the.

STSG, infection, and reintervention, defined as a return to OR for further surgery at the original wound site. A subanalysis of graft failure was conducted on PC wounds only.

**Results:** A total of 114 patients underwent STSG for 164 chronic LE wounds. The majority of wounds had PC (*n* = 128, 78.1%) while only 36 wounds (22.0%) had NC. Overall, the cohort had a median age of 63 (IQR: 19.5) and BMI of 29.4 (IQR: 9.3), with no differences between groups (*p* = 0.617 and *p* = 0.430, respectively). Charlson Comorbidity Index (CCI) for the PC group was 5 (IQR: 3) and 4 (IQR: 3.5) for the NC group (*p* = 0.212). On the date of STSG, median wound sizes for the PC wounds were larger than NC groups (23.3, IQR: 48.3 cm^2^ vs. 38, IQR: 109.5 cm^2^, *p* = 0.204). Of the PC wounds, 63.3% (n = 81/128) were polymicrobial, 74.2% (*n* = 95/128) contained gram positive organisms, and the majority (51.2%) were quantified as “light” volume of growth. There were no differences in graft failure (18.8% vs. 19.4%, *p* = 0.925) or reintervention (38.4% vs. 22.2%, *p* = 0.073) between the PC and NC groups. However, PC wounds demonstrated a significantly higher rate of infection (17.2% vs. 2.8%, *p* = 0.028) compared to NC wounds. Subanalysis of PDC wounds demonstrated that wounds that had heavy bacterial loads (*p* = 0.035) and were polymicrobial (*p* = 0.024) had significantly higher rates of graft failure.

**Conclusions:** Our findings suggest that the presence of a PC prior to STSG placement for chronic LE wounds does not adversely affect postoperative outcomes when compared to NC. Rather, it is the quality of the PC, specifically polymicrobial presence and heavy growth, that significantly influences the outcome of the graft. This challenges the conventional emphasis on achieving negative cultures before proceeding with STSG. We show that a positive culture result alone does not necessarily impact outcomes.

## 46 THOUGHTFUL OUTFLOW USING A “VASCULOPLASTIC APPROACH”: THE IMPORTANCE OF PREOPERATIVE VENOUS TESTING IN PLANNING FOR LOWER EXTREMITY FREE TISSUE TRANSFER

### 46.1 **Authors:** Monique Bautista Neughebauer, Georgetown University School of Medicine; Karen Li, Georgetown University School of Medicine; Christian Lava, MS, MedStar Georgetown University Hospital; Brain Truong, Georgetown University School of Medicine; Karen Evans, MD, MedStar Georgetown University Hospital

**Background:** Characterizing venous outflow is critical yet understudied in atraumatic lower extremity (LE) free tissue transfer (FTT) such as those performed in diabetics. Our study sought to investigate the utility of preoperative venous testing in LE FTT.

**Methods:** A retrospective review of patients at a single‐institution that underwent LE venous duplex imaging FTT for LE wounds were included in this study between 2011 and 2023. LE venous duplex findings, surgical details, comorbidities, and outcome data were collected. The presence of venous reflux (VR) and venous thrombosis (VT) were characterized in this patient population.

**Results:** Among patients who underwent FTT and preoperative venous US duplex testing, venous thrombosis was detected in 16.9% (*n* = 39/231) of the patient population. We found the rate of acute VTs to be 4.3% and chronic VTs to be 9.5%. Older age (OR = 1.07, CI = 1.00–1.13) and history of VT (OR = 6.58, CI = 2.11–20.53) remained independently predictive of VT on venous testing on multivariate analysis. Separately, our study detected the presence of VR in 75.1% (*n* = 136/181). VR group saw higher rates of immediate flap success (100% vs. 93.3%, *p* = 0.002) but no significant differences in rates of postoperative ipsilateral amputation.

**Conclusions:** A multidisciplinary “vasculoplastic” approach with plastic and vascular surgery to complex limb salvage is important. The utility of preprocedural venous testing helps detect abnormalities that may threaten flap success and guide the decision‐making in choosing the healthy venous system for microsurgical anastomosis. Further work needs to be performed to continue to understand outflow and the role of the venous system in lower extremity free tissue transfer.

## 47 TWIST, TURNS, AND TIBIAS: NAVIGATING TIBIAL AND FIBULAR MALUNION WITH ANKLE VARUS – A DECADE DEFERRED

### 47.1 **Authors:** Jeffrey Ng, DPM, Metropolitan Hospital Center; Johanna Godoy, DPM, Metropolitan Hospital Center

**Background:** Post‐traumatic arthritis with angulated deformity often results from intra‐articular and displaced fractures, leading to gait difficulties and instability, impacting adjacent or proximal joints. Traditional limb lengthening procedures may not fully address multi‐planar angulation deformity, underscoring the importance of considering the center of rotation of angulation (CORA) in multiple planes (i.e. sagittal and frontal). Neglecting this aspect can lead to ineffective limb lengthening and potential complications.

**Methods:** A 50‐year‐old diabetic patient presented with gait abnormalities and chronic pain due to a high‐impact fall a decade ago, with no prior surgical intervention for displaced fractures of the distal tibia and fibula. Comprehensive imaging (e.g., Radiographs, CT and Scanogram) revealed LLD and multi‐planar angulated deformity including tibial varum, rearfoot varum and tibial recurvatum. Surgical planning involved measuring coronal and sagittal CORAs (15° and 11°), followed by tendoachilles lengthening, tibiotalocalcaneal fusion, tib‐fib lengthening with an external fixator, and stabilization with an intramedullary rod.

**Results:** The procedure successfully corrected LLD (1.9 cm) and multi‐planar deformity, leading to a close‐to‐normal gait at the 6‐month follow‐up with no open lesions. Deformity corrections have been quantified and supported by radiographs and advanced imaging.

**Conclusions:** This showcases a limb deformity correction approach considering an extra plane for comprehensive LLD resolution. The encouraging outcome merits further studies, suggesting its potential effectiveness for patients with combined LLD and angulated deformities.

## 48 USE OF NOVEL CUSTOM OFFLOADING ANKLE FOOT ORTHOSES IN THE REDUCTION OF DIABETIC FOOT ULCERATIONS

### 48.1 **Authors:** Britain Wetzel, DPM, Johns Hopkins Hospital; Alexander Lakner, DPM, Private Practice; Lourdes Princess‐Filippi, MSOTR/L, CLT, Johns Hopkins Hospital; Ronald Sherman, DPM, Johns Hopkins Hospital

**Background:** Midfoot and rearfoot diabetic foot ulcerations are both limb and life‐threatening wounds. While they are inherently difficult to heal, offloading and reduction of motion in these areas are pillars for limb salvage efforts. Modalities such as external fixation, total contact casting, and keeping patients non‐weightbearing are often utilized for these types of wounds, but they are not without risks in insensate patients. The purpose of our study was to determine if this novel AFO could promote wound healing in patients with diabetic foot ulcers.

**Methods:** A retrospective analysis was performed on our patients who had a custom AFO fabricated to offload their diabetic ulcerations. The Multidisciplinary Diabetic Foot and Wound Care (DFWC) team collaborated with occupational therapy to create a thermoformed brace based off the design of a “sugar tong” orthosis generally utilized for elbow and forearm splinting. The material is moulded to the areas of intact skin and a pocket is created to offload ulcerations. This brace can be utilized in both surgical flats and diabetic shoes.

**Results:** Within our patient population, there has been a positive correlation between the utilization of the custom offloading AFO and the reduction of wound size. Many patients have completely healed, and others are projected to be fully healed with additional follow up.

**Conclusions:** Our custom AFO devices have shown to be effective in the reduction of diabetic foot ulcerations. The devices are easy to fabricate and maintain. They allow for a cost‐efficient option for patients to ambulate safely and promote wound healing.

## 49 UTILIZING COMPUTER ASSISTED GAIT ANALYSIS (CAGA) FACILITATES DIABETIC ORTHOTICS REDUCING DIABETIC FOOT ULCERATIONS

### 49.1 **Authors:** Britain Wetzel, DPM, Johns Hopkins Hospital; Jay Segel, DPM, Segel Podiatry; Brian Murray, DPT, Johns Hopkins Hospital; Mark Hopkins, DPT, Johns Hopkins Hospital; Ronald Sherman, DPM, Johns Hopkins Hospital

**Background:** Abnormal gait parameters can be better identified by the use of dynamic gait testing, as opposed to traditional static casting. This data can then be translated to offer patients more effective ways to prevent ulceration. The use of CAGA technology to help further understand the temporospatial metrics seen in diabetic patients can allow clinicians to create and adjust orthotics to prevent the recurrence of ulceration.

**Methods:** Patients who had a history of healed diabetic foot ulcer(s) were considered for participation. Patients walked on the Noraxon treadmill barefoot and with their normal shoes for initial gait analysis. Based on their gait pattern, a customized insert was fabricated to use in standardized diabetic shoes. Patients then followed up for re‐testing in their inserts and standardized shoes.

**Results:** 37 patients (*n* = 37) had computer assisted gait analysis barefoot, in normal shoes, and in customized orthotics in standardized shoes. Of our cohort, 2 patient re‐ulcerated during the study period. Of these patients, one patient reported that they had not been compliant with the use of orthotics, the other was determined to have ill‐fitting shoes that were not from the standardized shoe manufacturer.

**Conclusions:** Computer Assisted Gait Analysis (CAGA) has shown to have a strong correlation between the use of temporospatial analysis for the fabrication of custom orthotics and the prevention of re‐ulceration in our diabetic patients. As diabetics routinely have health checks for their eyes, HgbA1c, etc., a similar emphasis on analysing the gait metrics in this population may allow for more preventative measures to reduce ulcerations.

## 50 A NEW STANDARD OF CARE FOR DIABETIC PARTIAL FOOT AMPUTATION WOUND CLOSURE?

### 50.1 **Authors:** Matthew Regulski, DPM, Wound Care Institute of Ocean County NJ

**Background:** Diabetic partial foot amputations (toe, ray, TMA, etc) are known to have wound healing complication rates as high as 60%. Conventional sutures enclose and compress tissue, are particularly problematic for poorly perfused diabetic tissue, and may lead to ischemic necrosis and/or skin tearing (“cheesewiring”) of the skin. Adhesive suture retention devices (ASRD) are a new technology that shift suture tension away from the skin edge. In doing so, they protect skin edge perfusion and prevent skin tearing. They can be left in place for several weeks, providing the sustained mechanical support needed to heal diabetic wounds. Prior study has shown that ASRD reduce complications in diabetic amputations, including the need for revisional surgery, and this study sought to corroborate those findings.

**Methods:** Fifteen diabetic patients undergoing limb salvage underwent a variety of partial foot amputations (toe, ray, TMA, etc). ASRDs were used in the closure of all amputation wounds. ASRDs were left in place up to 6 weeks postop (note this is an off‐label use as IFU for ASRD recommends 2 weeks of use postop).

**Results:** When compared to historical controls, marked improvements were noted in surgical outcomes, including reductions in wound dehiscence and the need for revisional surgery.

**Conclusions:** This experienced limb salvage surgeon has since adopted the use of ASRD on all diabetic partial foot amputations and suggests that they should be considered as a new standard of care for wound closure in this high‐risk group of patients.

